# A novel inflammation-related lncRNAs prognostic signature identifies LINC00346 in promoting proliferation, migration, and immune infiltration of glioma

**DOI:** 10.3389/fimmu.2022.810572

**Published:** 2022-10-13

**Authors:** Wen-Jing Zeng, Lei Zhang, Hui Cao, Dongjie Li, Hao Zhang, Zhiwei Xia, Renjun Peng

**Affiliations:** ^1^ Department of Pharmarcy, Xiangya Hospital, Central South University, Changsha, China; ^2^ Department of Neurology, Beijing Hospital, National Center of Gerontology, Institute of Geriatric Medicine, Chinese Academy of Medical Science, Beijing, China; ^3^ Department of Psychiatry, The Second People’s Hospital of Hunan Province, The Hospital of Hunan University of Chinese Medicine, Changsha, China; ^4^ Department of Geriatrics, Xiangya International Medical Center, Xiangya Hospital, Central South University, Changsha, China; ^5^ Department of Neurosurgery, Xiangya Hospital, Central South University, Changsha, China; ^6^ Department of Neurology, Hunan Aerospace Hospital, Changsha Medical University, Changsha, China; ^7^ National Clinical Research Center for Geriatric Disorders, Xiangya Hospital, Central South University, Changsha, China

**Keywords:** glioma, lncRNA, inflammation, prognostic, target

## Abstract

In this study, a total of 13 inflammation-related lncRNAs with a high prognostic value were identified with univariate, multivariate Cox regression analysis, and LASSO analysis. LINC00346, which is one of the 13 lncRNAs identified, was positively associated with type 2 macrophage activation and the malignant degree of glioma. Fluorescence *in situ* hybridization (FISH) and immunohistochemical staining showed that LINC00346 was highly expressed in high-grade glioma, while type 2 macrophages key transcription factor STAT3 and surface marker CD204 were also highly expressed simultaneously. LINC00346 high-expression gliomas were more sensitive to the anti–PD-1 and anti-CTLA-4 therapy. LINC00346 was also associated with tumor proliferation and tumor migration validated by EdU, cell colony, formation CCK8, and transwell assays. These findings reveal novel biomarkers for predicting glioma prognosis and outline relationships between lncRNAs inflammation, and glioma, as well as possible immune checkpoint targets for glioma.

## Introduction

Glioma is the most frequent adult malignant brain tumor in the central nervous system. Gliomas are classified into grades I–IV based on the degree of malignancy; grades I and II tumors are regarded low-grade gliomas, while grades III along with IV tumors as high-grade gliomas, with glioblastoma (GBM) being the most aggressive malignant tumor type (1). Despite tremendous improvements in surgical resection approaches, radiotherapy, along with chemotherapy, the median survival time in individuals with GBM receiving these forms of treatment is 15 to 18 months (2, 3). So, it is pivotal to specify novel biomarkers and strategies for the diagnosis and effective treatment of malignant gliomas.

Epidemiological studies showed that a quarter of tumors are remarkably linked to inflammation (4). Inflammation enhances the onset and progress of cancer through complicated physiological along with biochemical processes (5, 6). Recent investigations regarded glioma, especially GBM, as a type of cancer strongly associated with inflammation status and immune responses (5). Regarding high-grade gliomas, the brain tissue is infiltrated with numerous immune cells, consisting of macrophages, microglia, neutrophils, and eosinophils (4). Stimulation of the immune cells by various signaling factors leads to intracellular oxidative stress and cytokine-mediated cascades of inflammation, causing DNA damage along with diminished DNA repair. This then drives subsequent mutations and contributes to additional mutations as well as epigenetic alterations as glioma cells progress (7, 8). In addition, chemokines, cytokines, and growth factors secreted within the tumor microenvironment polarize M1 to the immunosuppressive M2 phenotype, contributing to the glioma’s progression (9). Compared with glioma patients with higher levels of M2 macrophage, patients with elevated levels of M1 macrophage had better survival (10). Even though some research progress has been made in exploring the relationship between inflammation and gliomas (3, 11) further studies are needed to provide insights on how inflammation associates with gliomas and specify the novel biomarkers and therapeutic targets for gliomas.

Whole-genome sequencing reveals that more than 90% of the human genome is transcribed, but only approximately 2% of transcribed RNA is translated into protein (12). The remaining portion of genome mainly encodes non-coding RNA, particularly lncRNAs (13, 14). LncRNAs constitute nonprotein coding transcripts consisting of more than 200 nucleotides. Previously, lncRNAs were often considered as transcriptional “noise” (15). However, recent studies reported that several lncRNAs have other critical functions. They function through the competitive binding of molecules such as miRNAs (16). Studies reported that aberrant lncRNA expression in a surgical glioma section is implicated in glioma development by regulating cell proliferation, apoptosis, GSC self-renewal, differentiation, and inflammation (17–19). Bioinformatics analysis found that lncRNA expression patterns in clinical glioma specimens correlated with histological differentiation and malignancy grade, which may have remarkable clinical impacts for glioma sub-classification, diagnosis, and prognostication (20). Other studies found that RP11-732M18.3 was highly overexpressed in glioma cells, which not only promote glioma angiogenesis by accelerating the transcription and secretion of VEGFA but also facilitate glioma growth through accelerating p21 degradation (21, 22). More recently, a risk model of constructed from eleven inflammation-related lncRNAs was reported as a potential prognostic biomarker for patients with lower-grade gliomas (23). However, systematic studies on the relationship between inflammation-related lncRNAs and glioma prognosis remain unclear.

Herein, inflammation-related lncRNAs with prognostic significance were screened to estimate the survival of individuals with gliomas. Gene expression data coupled with the matching clinical data of individuals with gliomas were abstracted from two data resources: The Cancer Genome Atlas (TCGA), as well as the Chinese Glioma Genome Atlas (CGGA). A total of 13 prognostic lncRNAs related to inflammation were identified utilizing univariate and multivariate analysis and lasso regression analysis, and a risk scoring model was subsequently established. Gene Ontology (GO) explorations were employed to clarify the biological functions and the mechanisms relevant to lncRNAs. Gene set enrichment analysis (GSEA) was carried out to determine remarkably enriched cascades as per the risk scores. FISH, immunohistochemical staining, and FASC were performed to explore the relationship between LINC00346 and macrophages. CCK8, 5-ethynyl-2’-deoxyuridine (EdU), colony formation, and transwell assays were utilized to explore the tumor-promoting role of LINC00346. These data will greatly help to the precise diagnosis and individualized treatment for gliomas patients.

## Methods

### Data resources

Gene expression profile and the matched clinical data were abstracted from the TCGA data source (https://xena.ucsc.edu) and the CGGA data resource (http://www.cgga.org.cn). The TCGA cohort served as the training set, while the CGGA dataset served as the validation set. A total of 645 samples were obtained from the TCGA (LGG samples 508 and GBM samples 137) and 306 samples from CGGA (LGG samples 169 and GBM samples 137). The gene sets related to inflammation were obtained using the Molecular Signatures Database (MSIGDB) (https://www.gsea-msigdb.org/gsea/msigdb). Somatic mutation and copy number variation (CNV) data of individuals with gliomas were obtained from the TCGA database.

### Screening lncRNAs associated with prognosis and inflammation lncRNAs

Firstly, inflammatory-related lncRNAs were obtained through correlation analysis using GO (biological process) terms of inflammatory-related gene sets. Univariate Cox regression was used to specify lncRNAs linked to the survival of glioma patients. Subsequently, multivariate Cox regression analysis was carried out to determine lncRNAs with independent prognostic significance. LASSO regression analysis was then performed to identify the signature lncRNAs. A set of predictive lncRNAs along with their regression coefficients were determined (β).

### Consensus clustering of lncRNAs

To explore the role of the 13 identified lncRNAs in gliomas, glioma patients were clustered into different groups using the R “ConsensusClusterPlus” package. Survival analyses were performed with the R “survival” package.

### Clustered genomic alterations

To assess the connection between the risk score and the genomic features in gliomas, CNV coupled with somatic mutation assessments were conducted on the basis of the TCGA dataset. GSITIC assessment was carried out to elucidate genomic event enrichment.

### Construction of a prognostic nomogram on the basis of clinical features and risk score

We conducted univariate Cox regression on the risk score and clinical features with *P* < 0.0.5 as the threshold. Multivariate Cox analyses of the chosen characteristics were conducted, and a nomogram was created by the regplot package. Afterward, assessment of the risk nomogram was done with a calibration curve along with the AUC.

### Gene set variation analysis (GSVA)

The GSVA package was adopted to calculate the enrichment status of GO terms in TCGA and CGGA datasets. Correlation assessment was conducted between the risk score and GO terms, and items exhibiting *P* < 0.05 coupled with a high correlation coefficient were chosen (12). Correlation assessment between the risk score, and inflammation/immune cell type was conducted *via* the gene expression patterns from the TCGA datasets in R.

### Immunohistochemical staining

Glioma sections were obtained from the surgically resected glioma tissues of patients in Xiangya Hospital. Informed consent were obtained from all patients. All procedures were approved by the Ethics Committee of Xiangya Hospital, Central South University. Dewaxing of 5-µm-thick paraffin sections was done in xylene, followed by rehydration using different alcohol grades. Blocking of the activity of endogenous peroxidase was done *via* inoculation with H_2_O_2_ (0.3%). Afterward, we inoculated the sections with citrate buffer (0.1M; pH 6.0) followed by autoclaving for 3 min at 121˚C to enhance the accessibility of the antigen. Next, the sections were cooled and inoculated with H_2_O_2_ (0.3%) for 20 min to dampen the activity of endogenous peroxidase. Thereafter, rinsing of the sections in PBS (pH 7.2) was done and subsequently inoculated with antibodies (STAT3 and CD204; cat. no. sc-100627; Santa Cruz Biotechnology, Inc., United States). After that, the slides were inoculated with the secondary antibody (IgG) and rinsed in PBS, and the visualization of the peroxidase reaction was done *via* inoculation of the slides with DAB (0.02%), PBS (0.1%), and H_2_O_2_ (0.3%).

Finally, hematoxylin counterstaining was done, and subsequent dehydration in graded alcohol was performed and was then mounted in resin mount. Immunostaining results were evaluated separately by two independent pathologists.

### Cell culture and siRNA transfection

U87-MG and U251 cells, were supplied by Procell Life Science & Technology Co., Ltd (Hubei, China) and inoculated in DMEM (Sigma, USA) with 10% FBS (Gibco, USA) as well as 1% penicillin–streptomycin. The cells were grouped as follows: control group, siRNA-NC group, and LINC00346-siRNA group. Si-RNA was purchased from HonorGene (Changsha, China). About 5 µl siRNA and 5 µl lipofectamine 2000 (Invitrogen, USA) were diluted with a 95 µl serum-free medium. Then siRNA and lipofectamine were mixed and incubated at room temperature for 20 min. Finally, the mixed solution is introduced to each well of the culture plate containing the cells and the culture medium. The cells were transfected for 48 h and then harvested for subsequent experiments.

### Cell viability detection

Cell viability was detected by a CCK8 assay. The transfected cells were cultured in a 96-well plate for 24, 48, 72, and 96 h. Then, 100 μl of a medium containing CCK8 (Dojindo, Japan) was added to each well, and the optical density (OD) value at 450 nm was detected with a microplate reader (BioTek) after 4 h of incubation.

### Transwell migration assay

The cells were digested into single cells with trypsin and suspended in a serum-free medium, and then 100 µl of the cell suspension was introduced to the upper compartment of the transwell. About 500 µl of complete medium with 10% FBS was added to the lower chamber and the cells were incubated for 48 hours. Then, washed the upper chamber with PBS and wiped off the cells on the upper chamber. After fixing, the cells were stained with crystal violet. It was then washed with PBS, the membrane was placed on a glass slide, and observed under an inverted microscope. After decolorization with 10% acetic acid, the OD value at 550 nm was detected.

### Colony formation assay

The cells were resuspended and planted in six-well plates. After 2–3 weeks of incubation, the culture medium was discarded, and fixed the cells with 4% paraformaldehyde. Then the cell clones were dyed with crystal viole for 30 minutes at room temperaturet. The staining solution was washed off, and a picture of the colony was taken. After decolorization, the OD value at 550 nm was measured.

### Cell proliferation test

The EdU (5-ethynyl-2’-deoxyuridine) assay was used to evaluate cell proliferation (RiboBio, China). The cells were incubated overnight with 100 µl 50 μM EdU medium and then fixed with 4% paraformaldehyde. Then 100 µl 1×Apollo^®^ staining reaction solution was added and incubated for 30 min. After washing with 0.5% TritonX-100, the cells were incubated with 100 µl 1 × Hoechst 33342 reaction solution for 30 min. Finally, the cells were observed with a confocal microscope and pictures were taken. The cell proliferation rate was then calculated.

### Statistical analysis

R software was used for all statistical analyses. Remarkable differences between and among groups of normal distributed variables were measured using t-test or one-way ANOVA, respectively. Significant differences between and among groups of abnormal distributed variables were measured by Wilcoxon test or Kruskal-Wallis test, respectively. The chi-square test was application to the categorical data. The overall survival analysis was conducted with the Kaplan–Meier approach, and Cox regression was carried out using the R survival package. The gene set variation analysis (GSVA) package was adopted to compute the enrichment status in GO (Biological Process) (12). The R survival ROC package was adopted to create and visualize receiver operating characteristic (ROC) curves and compute the area under the curve (AUC) (13). All the statistical analyses were implemented in R. Somatic mutations and somatic copy number alternations (CNAs) data were abstracted from the TCGA data resource. *P*< 0.05 means statistically significant.

## Results

### Identification of prognostic lncRNAs related to inflammation

**Figure 1:**
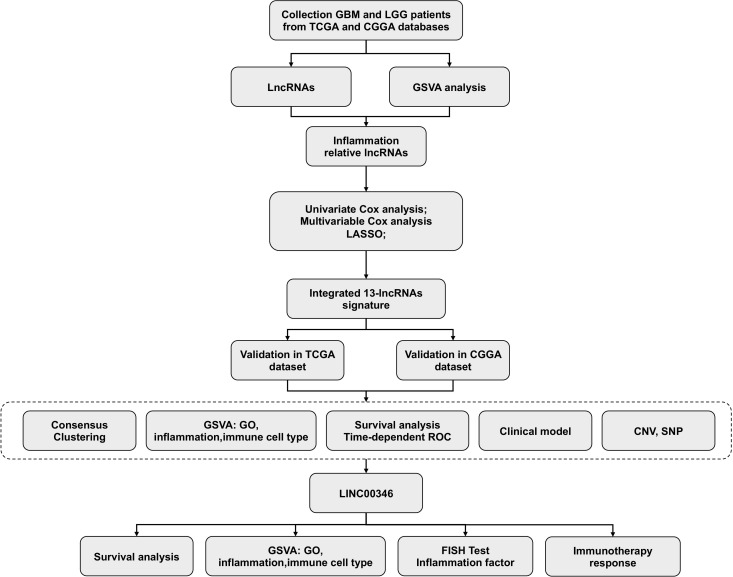
Flow chart of the whole research process.

**Figure 2:**
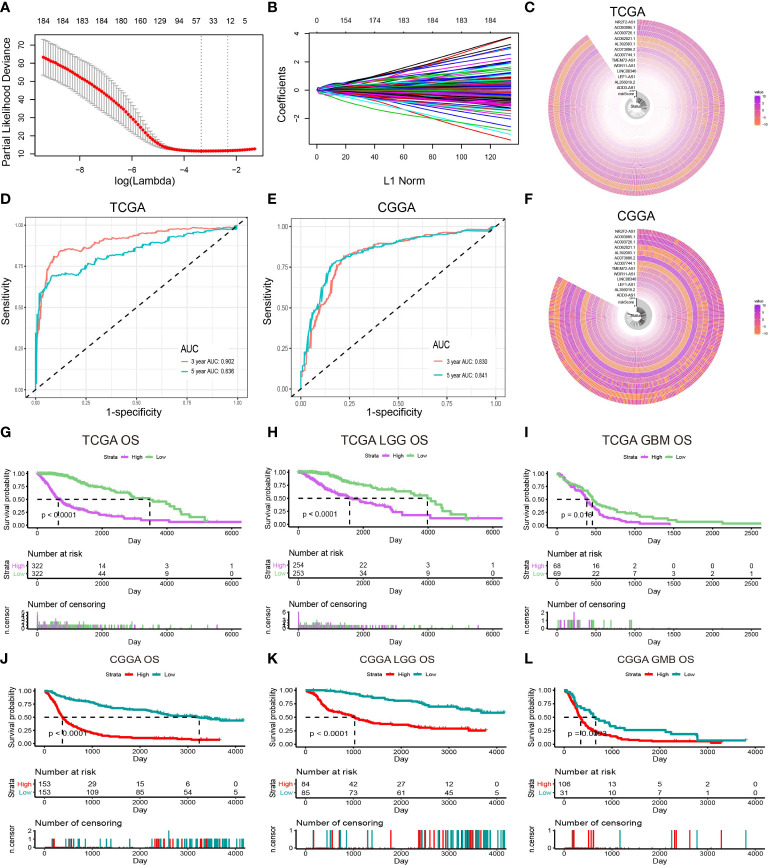
Identification of prognostic lncRNAs related to inflammation. **(A)** Ten-time cross-validation for tuning parameter selection in the LASSO model. **(B)** LASSO coefficient profiles of 13 prognostic lncRNAs. **(C)** The expression of prognostic lncRNAs in the TCGA dataset. **(D)** The ROC of the risk score model for predicting the 3- and 5-year survival of glioma patients in the TCGA dataset. **(E)** The ROC of the risk score model for predicting the 3- and 5-year survival of glioma patients in the CGGA dataset. **(F)** The expression of prognostic lncRNAs in the CGGA dataset. **(G–I)** Kaplan–Meier curves for overall survival (OS) between patients with high and low risk in the TCGA dataset. **(J–L)** Kaplan–Meier curves for OS between patients with high and low risk in the CGGA dataset.

As shown in **Figure 1**, 13,895 lncRNAs were obtained by intersecting the lncRNAs in TCGA and CCGA datasets. Univariate and multivariate Cox regression analyses were performed to explore the relationship of the patients’ disease-specific survival (DSS) with different lncRNAs expression levels. Univariate Cox analysis recognised 286 lncRNAs and multivariate Cox analysis identified 184 lncRNAs, which were deemed to have remarkable prognostic capacity at *P*-value < 0.05. Exploring interpretable estimation rules in high-dimensional data using lasso regression. Finally, 13 inflammation-related lncRNAs (ADD3-AS1, AL356019.2, LEF1-AS1, LINC00346, WDR11-AS1, TMEM72-AS1, AC007744.1, AC073896.2, AL392083.1, AC062021.1, AC093726.1, AC093895.1, and NR2F2-AS1) with prognostic values were identified using LASSO regression in TCGA dataset (**Figures 2A, B**). **Figures 2C, F** exhibited the expression of inflammation-related lncRNAs in the TCGA and CCGA datasets, respectively. To verify the sensitivity and specificity of the prognostic model based on the 13 inflammation-related lncRNAs, the AUC and ROC were determined. AUC values for 3- and 5-year in the TCGA dataset were 0.902 and 0.836, respectively, and the 3- and 5-year in the CGGA dataset were 0.830 and 0.841, respectively (**Figures 2D, E**), indicating that the risk score model showed good accuracy. In the TCGA cohort, there were significant differences in the risk score between the patients separately stratified by 1p/19q status, grade, *IDH* mutation status, *MGMT* promoter status, and molecular subtypes, but not by gender. Glioma patients with the classical and mesenchymal molecular subtype, *IDH*-wild type, 1p/19q non-co-deletion status, and *MGMT* promotor unmethylation showed higher risk scores (**Figure S1**). In addition, the risk score escalated with the increasing of WHO grade in gliomas. These results indicated that risk score was important in predicting the clinical–pathological characteristics of glioma.

The overall survival (OS) of the total, LGG, and GBM patients from the TCGA dataset are shown in **Figures 2G–I**. Patients were stratified into high- and low-risk groups based on the median risk score. There was a statistically remarkable difference in OS between high and low-risk groups and similar results were found in the CGGA cohort (**Figures 2J–L**). The disease-specific survival (DSS) and the progression-free interval (PFI) of patients in the high- and low-risk groups were also analyzed, and the results showed significant differences (**Figures S2A–F**).

### Consensus clustering of 13 prognostic inflammatory-related lncRNAs signature

On the basis of the expression similarity, the clustering stability of the TCGA dataset rising from k=2 to 10 was shown (**Figures S3A, S4A, B**). When k=2, the relative change in the area under the CDF indicated a flat middle segment (**Figure S4A**). Therefore, k=2 was selected as an adequate choice. Heatmap of the consensus matrix for k = 2 in the TCGA dataset is exhibited in **Figure S4C**. The predictive genes classified two clusters of glioma patients in the TCGA dataset with distinct clinical outcomes and clinicopathological characteristics by consensus clustering. Between groups, survival probability and PCA distribution were clearly separated (**Figures S4D, S5A–C**), and similar results were found in the CCGA dataset (**Figures S3B, S4E, F, S5D–F**).

### The significant correlation between genomic alterations in inflammatory-related lncRNAs and the glioma risk model

**Figure 3:**
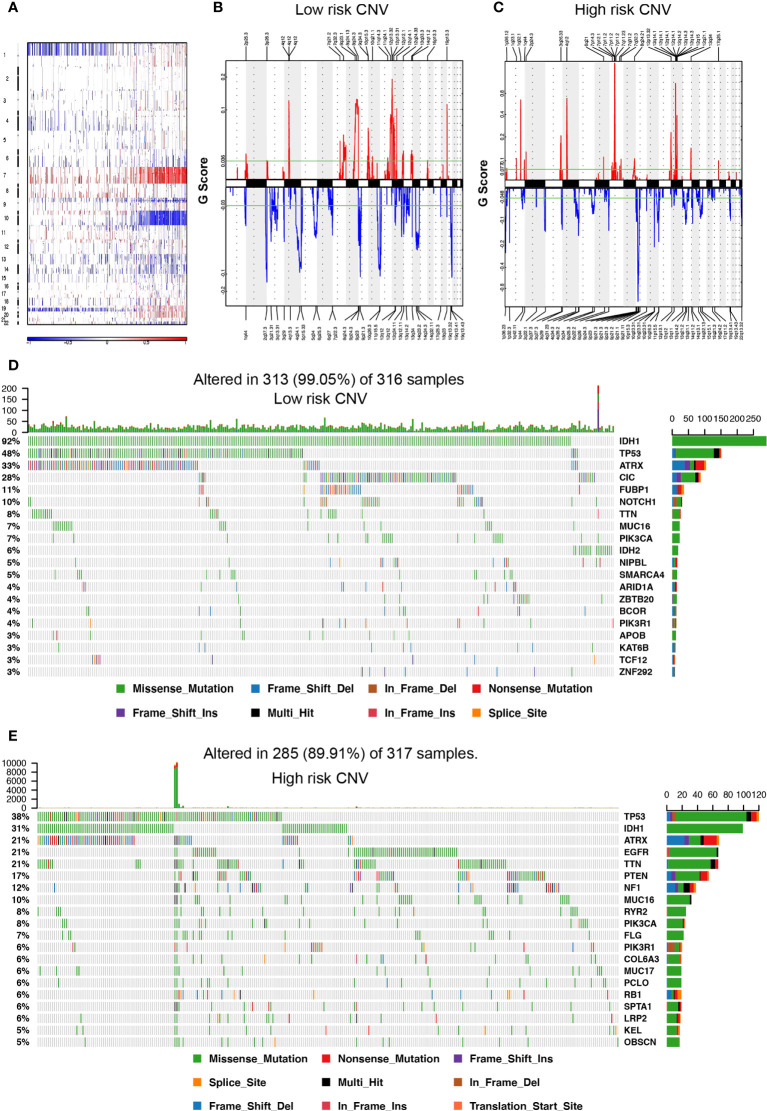
Somatic mutation and copy number variation (CNV) in the high- and low-risk score groups. **(A)** CNV in autosomes of glioma samples. **(B, C)** The distribution of CNV in autosomes in the high- and low-risk score group, respectively. **(D, E)** The waterfall plot displays the somatic mutation distribution of the top 20 most frequently mutated genes in the low- and high-risk groups, respectively.

Somatic mutation and CNV analysis demonstrated remarkable differences between the high- and low-risk groups. Considering the somatic copy number alternations’ functions in oncogenesis, the CNV between low- and high-risk samples was explored. In gliomas, the incidence of Chr 7 amplification and Chr 10 deletion escalated as the risk score increased, while the incidence of 1p/19q codeletion reduced with the risk score (**Figure 3A**). The GISTIC 2.0 assessment uncovered numerous remarkable amplified regions containing multiple oncogenes in glioma patients with higher risk scores, consisting of 1q32.1 (*PIK3C2B*), 12q14·1(*CDK4*), 7p11·2 (*EGFR*), and 4q12 (*PDGFRA*). Focal deletion peaks were detected in the high-risk group, for instance, 9p21·3 (*CDKN2A*), 10q23·31 (*PTEN*, *KLLN*), and 10q26·3 (*DUX4*). The focal amplification and deletion peaks also presented in the patients with low risk scores, their G values were significantly lower. Moreover, there were remarkable amplification regions (19p13.3, 12q14.1, 11q24.1, and 4q12) and deletion (9p21.3, 11p15.5, 4q34.1, and 10q26.3) reported gliomas with low-risk score groups (**Figures 3B, C**). Somatic mutations presented in 285 (89.91%) and 313 (99.05%) of samples in the high- and low-risk groups, respectively. The mutation frequencies of *IDH1* and *ATRX* in gliomas with a low-risk score were remarkably higher than in gliomas with a high-risk score (*IDH1*, 92% vs. 31%; *ATRX*, 33% vs. 21%), while *TTN* and *MUC16* had lower mutation frequencies (*TTN*, 8% vs. 21%; *MUC16*, 7% vs. 10%). *EGFR* (21%), *PTEN* (17%), and *NF1* (12%) mutations in the high-glioma risk score group and *CIC* (28%), *FUBP1* (11%), and *NOTCH1* (10%) mutations in the low-risk score group were identified (**Figures 3D, E**), and the mutation frequency of these genes was greater than 10%.

### Functional analysis of the prognostic inflammatory-related lncRNAs signature

**Figure 4:**
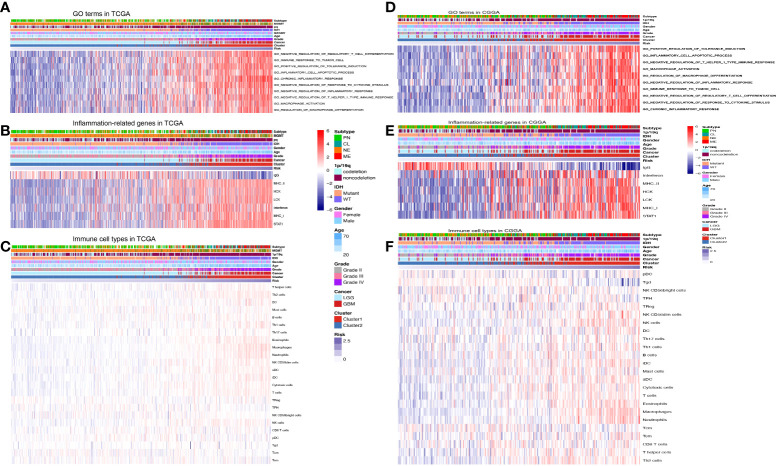
Functional analysis of the prognostic inflammatory-related lncRNAs signature. **(A)** Representative GO terms significantly correlated with the prognostic lncRNAs signature in the TCGA dataset. **(B)** Inflammation genes correlated with the prognostic lncRNAs signature in the TCGA dataset. **(C)** Immune cell types correlated with the prognostic lncRNAs signature in the TCGA dataset. **(D)** Representative GO terms significantly correlated with the prognostic lncRNAs signature in the CGGA dataset. **(E)** Inflammation genes correlated with the prognostic lncRNAs signature in the CGGA dataset. **(F)** Immune cell types correlated with the prognostic lncRNAs signature in the CGGA dataset.

To further assess the function of the 13 prognostic inflammatory-related lncRNAs signature, GO and GSVA analyses were performed with TCGA and CGGA datasets. The top enriched lncRNAs functions included negative modulation of regulatory T-cell differentiation, the immune response to the tumor cell, positive modulation of tolerance induction, inflammatory cell apoptotic process, chronic inflammatory response, negative regulation of response to cytokine stimulus, macrophage activation, and regulation of macrophage differentiation (**Figures 4A, D**). Even though the orders of each enriched lncRNAs function in the TCGA and CCGA datasets were different, their overall correlation with immune function was similar. Further analyses of inflammation genes correlated with the prognostic inflammatory-related lncRNAs signature in the TCGA and CCGA datasets showed that the genes IgG, interferon, MHC-II, HCK, LCK, MHC-I, and STAT1 were the most related to the 13 prognostic inflammatory-related lncRNAs signature (**Figures 4B, E**). To further detect whether the 13 prognostic inflammatory-related lncRNAs signature affected the immune cells, the types of immune cells involved in gliomas were analyzed, and it was found that the T helper cells, NK cells, DC, macrophages, etc., are all involved in gliomas (**Figures 4C** **, F**). Immunity correlation analysis showed that macrophages, neutrophils, eosinophils, and Th2 cells had the highest correlation with the selected lncRNAs in the TCGA and CCGA datasets (**Figure S6**). This indicated that the 13 prognostic inflammatory-related lncRNAs signature may participate in immune responses in glioma progression. We created a ceRNA regulatory network through the 13 lncRNAs (**Figure S7**).

### Prognostic nomogram for overall survival prediction

**Figure 5:**
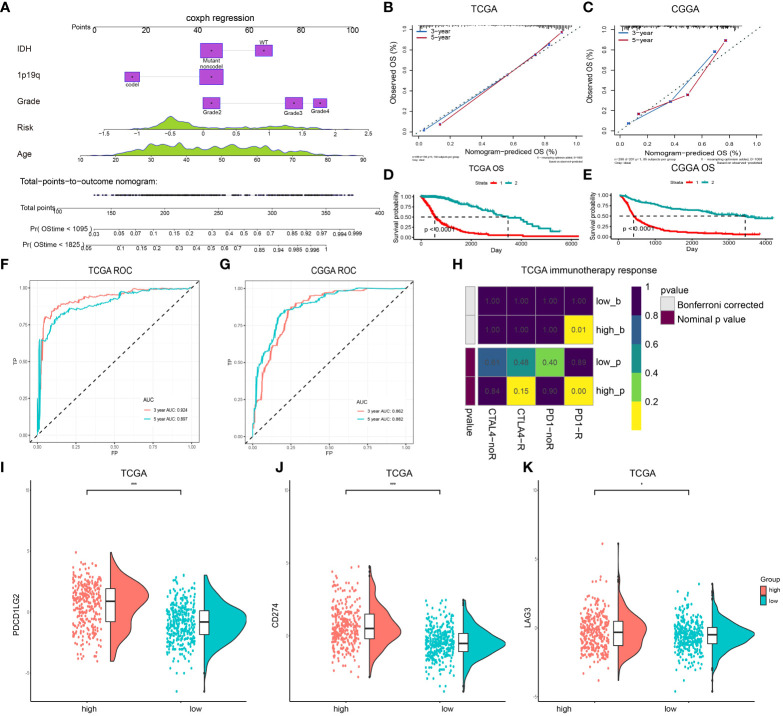
A nomogram for predicting OS of patients in the TCGA dataset. **(A)** The nomogram is created by adding points identified on the points scale for each variable. **(B, C)** Internal calibration curve for validation of the nomogram model in the TCGA and CCGA datasets. **(D)** Kaplan-Meier curves for OS between patients with the high risk and the low-risk of the nomogram model in the TCGA datasets. **(E)** Kaplan-Meier curves for OS between patients with the high risk and the low-risk of the nomogram model in the CGGA dataset. **(F, G)** The ROC for predicting the 3- and 5-year survival of glioma patients in TCGA and CCGA datasets. **(H)** Submap analysis of CTLA4 and PD-1 treatment in the high- and low-risk groups. **(I–K)** Expression level of PDCD1LG2 **(I)**, CD274 **(J)**, and LAG3 **(K)** in the high- and low-risk groups. (***P < 0.001).

To determine if this prognostic classifier could perform as an independent indicator in gliomas, a nomogram that integrated lncRNAs classifiers and clinicopathological characteristics, including age, *IDH* mutation, 1p/19q, and the WHO grade, was constructed to estimate the 3- and 5-year survival rate in glioma patients (**Figure 5A**). The calibration curve exhibited that the estimated 3- and 5-year survival rates were remarkably correlated with the observed ratio in the TCGA and CCGA datasets (**Figures 5B, C**). These results demonstrated that lncRNA signatures' stability and predictive performance are superior to multiple clinical features. As shown in **Figure 5D**, OS differences between the high- and low-risk groups were statistically significant (*P* < 0.05). Similar results were found in the CGGA dataset (**Figure 5E**). Then, the AUC and the ROC were determined and the 3- and 5-year survival rates were compared. The risk scoring model was found to exhibit good accuracy (**Figures 5F, G**). Decision curve analysis (DCA) was employed to assess the risk score model. DCA results for 5-year survival predictions showed that the multifactor model prognostic estimation based on the lncRNAs added more net benefit than the “*IDH-*only” or “grade-only” strategies in the TCGA datasets (**Figure S8**). To assess whether immune therapy deserved to be tried, the TIDE online database was utilized to predict the response of CTLA4 and PD-1 treatment in high- and low-risk gliomas. Significant differences in PD-1 treatment effects were observed between high- and low-risk gliomas (**Figure 5H**), meaning that PD-1 may be a potential immune therapy target for high-risk gliomas. In addition, the expression level of PD-1 ligands CD274 and PDCD1LG2, as well as another immune checkpoint LAG3 were assessed, and the result suggested that LAG3 expression was increased in the high-risk group (**Figures 5I–K**). LINC00346 is associated with the prognosis of gliomas.

**Figure 6:**
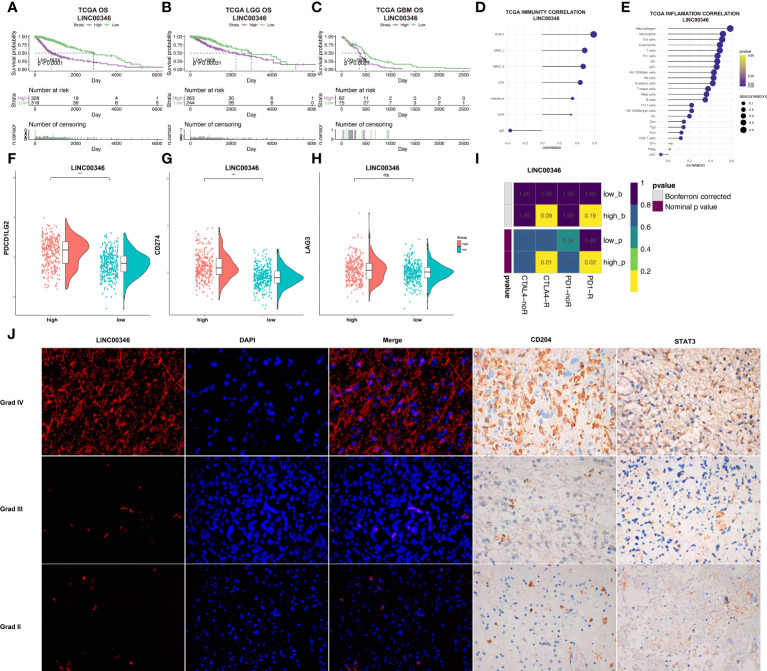
LINC00346 expression is associated with the prognosis of gliomas. **(A–C)** Differences in OS between high LINC00346 expression and low LINC00346 expression groups among total, LGG, and GBM patients in the TCGA dataset. Patients were divided into high LINC00346 expression and low LINC00346 expression groups based on the median of LINC00346 expression. **(D, E)** Immunity and inflammation related to LINC00346 expression **(D)** and immune cell types **(E)** and in the TCGA dataset. **(F–H)** Expression level of PDCD1LG2, CD274, and LAG3 between high LINC00346 expression and low LINC00346 expression groups (*** P < 0.001, ns, not significant). **(I)** Submap analysis manifested that LINC00346 high-expression group could be more sensitive to the anti–PD-1 and anti-CTLA-4 therapy. **(J)** Dual color fluorescence *in situ* hybridization (FISH) was used to detect the expression of LINC00346 in grades II, III, and IV gliomas, and immunohistochemistry was used to detect the expression of CD204 and STAT3 in grades II, III, and IV gliomas.

LINC00346 was selected for further study. In the TCGA dataset, glioma patients were stratified into two groups based on the median expression level of LINC00346. The prognosis of the low LINC00346 expression group was remarkably higher relative to high LINC00346 expression group (*P* < 0.0001). Furthermore, survival analysis was carried out in sum, LGG, and GBM cohorts. In sum, LGG, and GBM cohorts, the OS, PFI, and DSS of the high LINC00346 expression group were worse than those of the low LINC00346 expression group (*P* < 0.05) (**Figures 6A–C**, **S9**). These findings indicated that LINC00346 is a poor prognostic indicator for gliomas.

Based on the possible association between lncRNA and inflammation reported in previous studies (24, 25) and our previous analysis, LINC00346 was suspected to be involved in inflammation based on its association with immune cells. LINC00346 was shown to be highly correlated with immune cells, especially macrophages, neutrophils, and Th2 cells (**Figures 6D, E**). GSEA showed that the high-expression LINC00346 subset was primarily linked to important inflammation-related hallmarks (**Figure S10**). In addition, the expression levels of CD274 and PDCD1LG2, but not LAG3, were found to be higher in the high LINC00346 expression group (**Figures 6F–H**). To investigate whether the expression of LINC00346 was correlated with immune checkpoint therapy, submap analysis of CTLA4 and PD-1 treatment in LINC00346 was performed in both high- and low-risk groups. As displayed in **Figure 6I**, LINC00346 high-expression patients may be more sensitive to the anti–PD-1 and anti-CTLA-4 therapy. Meanwhile, to explored whether LINC00346 correlated with the amount of M2 macrophage in gliomas with different grades. FISH experiment was utilized to detect the expression of LINC00346 in different WHO grades of gliomas, and immunohistochemical staining was utilized to investigate the M2 macrophage in gliomas with different WHO grades. M2 surface marker CD204 and key transcription factor STAT3 were used to indicate the M2 macrophage. The results indicated that LINC00346, CD204, and STAT3 were expressed higher in grade IV gliomas than in grade II gliomas (**Figure 6J**), suggesting that the amount of M2 macrophage in different grades of gliomas was positively correlated with the expression. Notably, in GBM and LGG, LINC00364 expression was obviously associated with the expression of ICPs, such as CXCL9, CD40, CD80, and CD28 (**Figure S11**). Therefore, the amount of M2 was positively related to the glioma grade, which was also consistent with the expression of LINC00346.

### LINC00346 affected the proliferation and migration of gliomas and regulated the differentiation of macrophages

**Figure 7:**
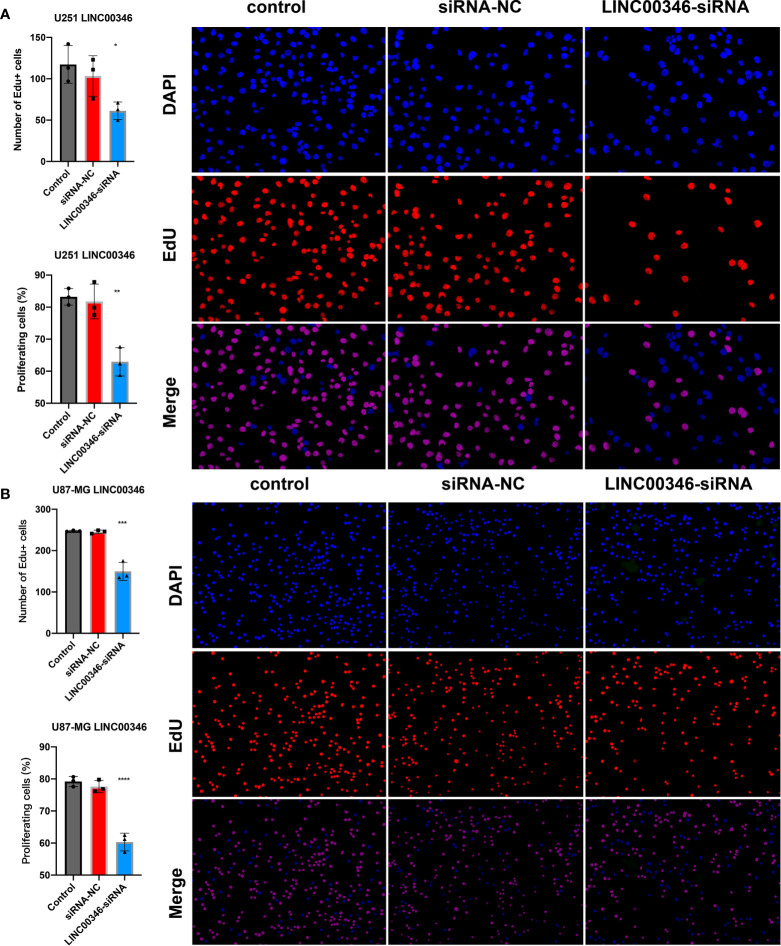
EdU assay was used to detect the proliferation of **(A)** U251 cells and **(B)** U87-MG cells. (*P < 0.05, **P < 0.01, ***P < 0.001, ****P < 0.0001).

**Figure 8:**
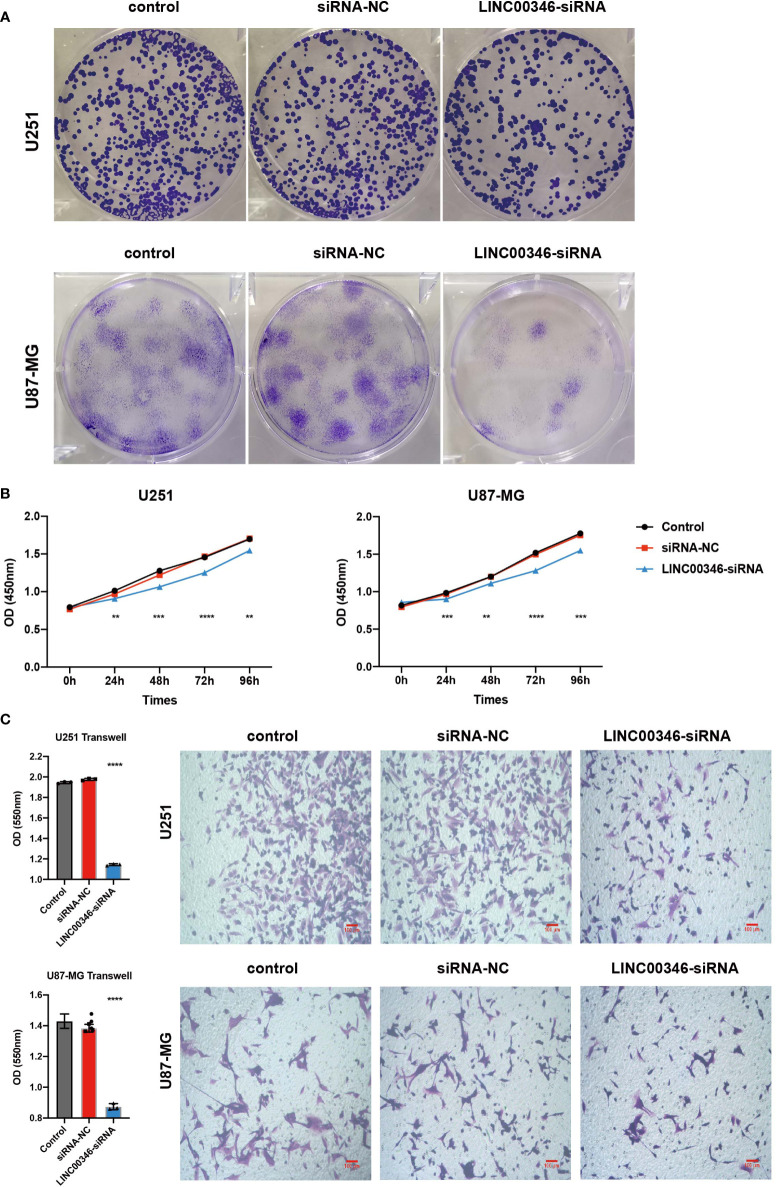
LINC00346 affected the proliferation and migration of glioma cells. After being transfected with siNC or siLINC00346, **(A)** cell colony assay and **(B)** CCK8 assay were used to detect the proliferation of U251 and U87-MG cells; **(C)** transwell assay was used to evaluated the migration of U251 and U87-MG cells. (**P < 0.01, ***P < 0.001, ****P < 0.0001).

**Figure 9:**
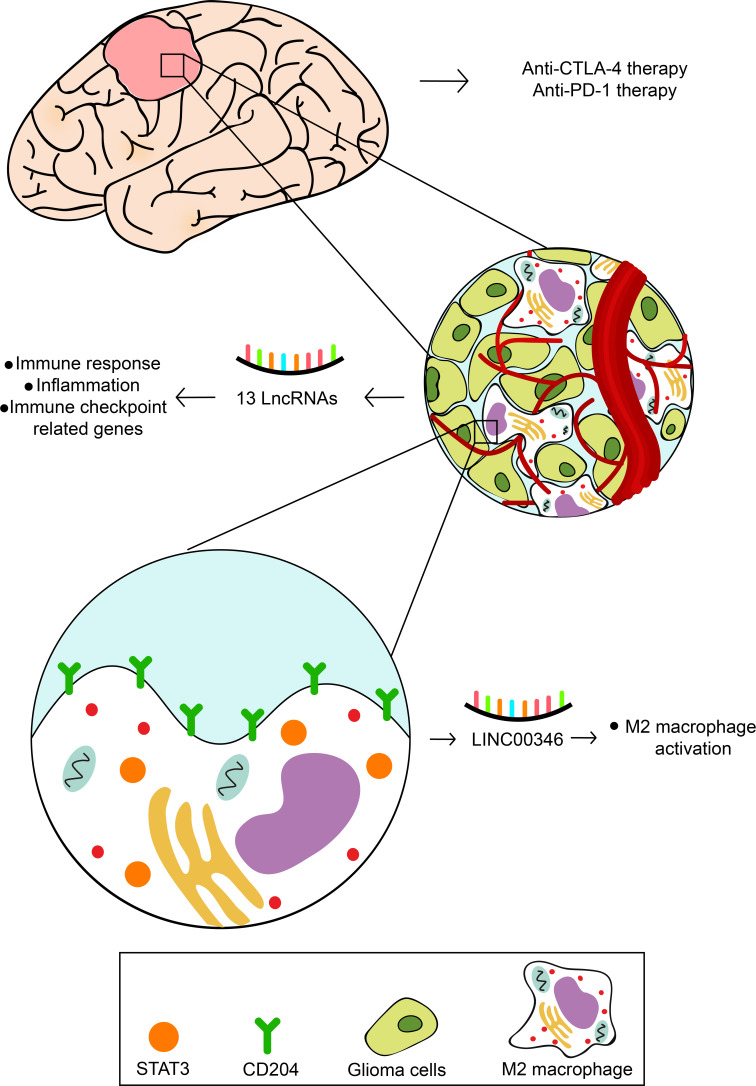
The speculative mechanism of 13 prognostic lncRNAs regulation glioma cells and immune cells.

To further explore the effect of LINC00346 on gliomas, we used glioma cell lines U251 and U87-MG to conduct further *in vitro* experiments. The inhibition of LINC00346 expression significantly reduced the proliferation of glioma cells in the EdU assay (**Figures 7A, B**). In addition, the colony formation assay also showed that knockdown of LINC00346 by siRNA significantly inhibited the viability of glioma cells (**Figure 8A**). The CCK8 assay showed that cell viability was inhibited by the silence of LINC00346 (**Figure 8B**). In order to further determine whether LINC00346 affects the metastasis of glioma, transwell migration assay was used to evaluate the migration ability of glioma cells. The results showed that inhibition of LINC00346 with siRNA significantly inhibited the migration of glioma cells (**Figure 8C**). Moreover, *in vitro* co-culture experiments were utilized to explore the role of LINC00346 in regulating macrophage differentiation and migration. Knockdown of LINC00346 expression in U87 and U251 cells obviously inhibited the migration of HMC3 cells (**Figure S12**). Above results proposed that the LINC00346 expression may accelerate in glioma progression through regulating glioma cell proliferation and migration (**Figure 9**).

## Discussion

Glioma is the most common malignant brain tumor in adults. Despite recent therapeutic advances against gliomas, the survival rate of glioma patients remains poor. Over the years, researches have been dedicated to study the link between inflammation and tumor growth. In glioblastoma patients, a more aggressive clinical course has been associated with Foxp3+ T regulatory cells, which mediate immune tolerance and inhibit antitumor immune responses (26). The immune checkpoint ligand programmed death-ligand 1 (PD-L1) functions as an immunoinhibitory molecule expressed in the tumor milieu and promotes glioma infiltration (27, 28). Although there are numerous studies on the relationship between inflammation and gliomas, there is still no proper treatment applied by clinics. Recently, lncRNAs have acquired increased attention, from initiation and development of tumors to inflammatory responses (25, 29). Some studies have reported that lncRNAs such as RP11-284N8.3.1 along with AC104699.1.1 can predict the prognosis and survival of ovarian cancer patients (24). However, studies on inflammation-related lncRNAs in gliomas remain unclear. This study found that 13 lncRNAs (ADD3-AS1, AL356019.2, LEF1-AS1, LINC00346, WDR11-AS1, TMEM72-AS1, AC007744.1, AC073896.2, AL392083.1, AC062021.1, AC093726.1, AC093895.1, and NR2F2-AS1), which were inflammation-related based on functional analysis, were differentially expressed in gliomas and normal brain tissue, and they could independently and accurately predict the prognosis of patients.

ADD3-AS1 is another transcript of ADD3 that encodes for Adducin Gamma and is implicated in the spectrin-actin network and present in the extrahepatic biliary epithelium. ADD3-AS1 encodes an lncRNA, which influences the expression of ADD3 (30). However, no studies have reported the involvement of ADD3-AS1 in gliomas. LEF1-AS1 is an lncRNA that acts as an oncogene in glioma. Silencing LEF1-AS1 expression inhibits GBM proliferation and invasion by reducing ERK, as well as Akt/mTOR signaling activities (31). LINC00346 has been documented to be overexpressed in non-small cell lung cancer, bladder cancer, and pancreatic cancer and serves as a positive transcriptional regulator of c-Myc, but its role in glioma has not been reported (32, 33). WDR11-AS1 (csf biomarker) modifies the association between tau positivity and neurodegeneration (34), and it is involved in thyroid cancer (35). TMEM72-AS1 and AC062021.1 are associated with the pathogenesis of major depressive disorder (MDD); however, dysregulated lncRNAs’ contribution to the development of MDD remains elusive (36, 37). AL392083.1 is involved in synergistic neurotoxicity (38). AC093726.1 is associated with breast cancer (39), while AC093895.1 plays a pivotal role in the modulation of cancer-related pathways (40). NR2F2-AS1 is involved in the regulation of cell cycle progression, cell apoptosis, and cell proliferation during cancer (41, 42). Patients harboring high-risk signatures had shortened overall survival, while patients harboring a low-risk signature had prolonged survival. Similar risk scores were obtained from cases in the TCGA and CGGA datasets and were used to confirm the prognostic values of the 13 lncRNAs in gliomas.

GSVA was conducted in the 13 prognostic inflammation-related lncRNAs signature to explore associated signaling cascades. GO analysis exhibited that the signature is mostly enriched in inflammation-related reactions. Inflammation has been linked to several tumors. Herein, the 13 lncRNAs primarily participated in the inflammatory response to the tumor and immune-linked molecules. In gliomas, remarkable efforts have been made over the years to establish the molecular signatures that may contribute to the diagnosis or treatment of patients. These studies have uncovered a series of molecular signatures that are linked to the prognosis of gliomas. *IDH1* mutation was added into the 2016 WHO glioma classification, and patients with this mutation develop poor prognosis compared with the *IDH* wild-type patients (1). Moreover, studies showed that genetic aberrations in these genetic markers might lead to remarkable epigenetic changes at the molecular level, including DNA methylation, mRNA expression, and lncRNA expression. This study found that inflammation-related lncRNAs may not only exist in associations with well-recognized genetic biomarkers but also provide new strategy into the prognosis and treatment of gliomas.

Among the 13 lncRNAs identified, LINC00346 was found to be the most associated with macrophages. In this study, LINC00346 expression in gliomas was determined by FISH, and type 2 macrophage surface marker CD204 and its key transcription factor STAT3 were determined by IHC staining. Three of the molecules detected were highly correlated with high-grade glioma. Studies have shown that STAT3 is a key transcription factor that supports macrophage differentiating into type 2 macrophage (43) and greatly contributes to tumor progression (9, 44). This study found that the high expression of LINC00346 is linked to the high expression of STAT3 and CD204, and LINC00346 may serve as a positive function in the STAT3 expression, enabling macrophages to differentiate into M2 and supporting the progression of gliomas. This study depicted the relationship between LINC00346 and macrophages. However, more detailed investigations are necessary to focus on finding the exact role of LINC00346 in gliomas, which may be to promote the progress of immunotherapy for gliomas. The targeting of immune checkpoints proved to be an efficient way to treat different tumors (45). In this study, the response of CTLA4 and PD-1 treatment in glioma was evaluated, and difference was found between high- and low-grade glioma groups, hinting that anti-CTLA4 or anti–PD-1 drugs might be a good method for high-grade glioma patients.

We selected LINC00346 in the feature lncRNA for further verification and analysis. We established that LINC00346 expression was negatively correlated with the clinical prognosis of gliomas. *In vitro* experiments, CCK8, and colony formation experiments displayed that silencing the expression of LINC00346 inhibits glioma cell viability. In the EdU test, the inhibition of LINC00346 expression repressed the growth of glioma cells. In addition, inhibition of LINC00346 interferes with glioma cell infiltration. In conclusion, knockdown of LINC00346 inhibited the viability, proliferation, migration, and invasion of glioma cells. This also confirms the result that we found that the inflammation-relative lncRNAs play an essential role in the onset and progress of gliomas.

Although our risk model has good performance in predicting the prognosis of glioma patients in the TCGA and CGGA cohorts, there are still many limitations. We should verify the differential expressions of these lncRNAs in glioma tissues and para-cancer tissues, as well as the prognostic value of the risk model with our own samples. The molecular mechanisms of these lncRNAs in gliomas and the efficacy of the risk model in clinical practice remain unclear and further experiments are needed.

## Conclusions

In conclusion, this study identified 13 inflammation-related lncRNAs signature that were linked to the survival of glioma patients through bioinformatic analysis. Moreover, the study also described the relationship between LINC00346 and macrophages. These findings may help in the development of efficient biomarkers for use in assessing the appropriateness of immunotherapy and potential implications in the diagnosis and treatment of gliomas.

## Data availability statement

The datasets used in this study are available in TCGA data source (https://xena.ucsc.edu) and CGGA data portal (http://www.cgga.org.cn).

## Author contributions

Conception and design, ZX, W-JZ and LZ; Foundation support, RP and ZX; Acquisition and analysis of data, W-JZ and LZ; Interpretation of data, W-JZ, LZ, HC, DL, and HZ; Drafting the manuscript and revising for submission quality, W-JZ, LZ, HC, DL and RP; Study supervision, RP; All authors contributed to the article and approved the submitted version.

## Funding

This work was supported by Science Foundation of the AMHT Group (No. 2020YK10) and National Natural Science Foundation of China (No. 81901268).

## Conflict of interest

The authors declare that the research was conducted in the absence of any commercial or financial relationships that could be construed as a potential conflict of interest.

## Publisher’s note

All claims expressed in this article are solely those of the authors and do not necessarily represent those of their affiliated organizations, or those of the publisher, the editors and the reviewers. Any product that may be evaluated in this article, or claim that may be made by its manufacturer, is not guaranteed or endorsed by the publisher.

## Abbreviations

lncRNAs, long non-coding RNAs; LGG, low-grade gliomas; GBM, glioblastoma; TCGA, The Cancer Genome Atlas; CGGA, Chinese Glioma Genome Atlas; GO, Gene Ontology; GSEA, gene set enrichment analysis; GSVA, gene set variation analysis; MSIGDB, Molecular Signatures Database; CNV, copy number variation; CNA, copy number alternation; OD, optical density; PBS, phosphate-buffered saline; EdU, 5-ethynyl-2’-deoxyuridine; FISH, fluorescence *in situ* hybridization; ROC, receiver operative characteristic; AUC, area under the curve; OS, overall survival; PFI, progression-free interval; DSS, disease-specific survival; DCA, decision curve analysis; PD-L1, programmed death-ligand 1; MDD, major depressive disorder.

## References

[1] Diamandis P Aldape K. World health organization 2016 classification of central nervous system tumors. Neurol Clin (2018) 36(3):439–47. doi: 10.1016/j.ncl.2018.04.003 30072064

[2] Woehrer A Bauchet L Barnholtz-Sloan JS. Glioblastoma survival: Has it improved? evidence from population-based studies. Curr Opin Neurol (2014) 27(6):666–74. doi: 10.1097/wco.0000000000000144 25364955

[3] Boussiotis VA Charest A. Immunotherapies for malignant glioma. Oncogene (2018) 37(9):1121–41. doi: 10.1038/s41388-017-0024-z PMC5828703 29242608

[4] Sowers JL Johnson KM Conrad C Patterson JT Sowers LC. The role of inflammation in brain cancer. Adv Exp Med Biol (2014) 816:75–105. doi: 10.1007/978-3-0348-0837-8_4 24818720

[5] Teng MW Galon J Fridman WH Smyth MJ. From mice to humans: Developments in cancer immunoediting. J Clin Invest (2015) 125(9):3338–46. doi: 10.1172/jci80004 PMC4588291 26241053

[6] Balkwill F Mantovani A. Inflammation and cancer: Back to virchow? Lancet (2001) 357(9255):539–45. doi: 10.1016/s0140-6736(00)04046-0 11229684

[7] Mantovani A Allavena P Sica A Balkwill F. Cancer-related inflammation. Nature (2008) 454(7203):436–44. doi: 10.1038/nature07205 18650914

[8] Peixoto E Khan A Lewis ZA Contreras-Galindo R Czaja W. The Chromatin Remodeler Hells: A New Regulator in DNA Repair, Genome Maintenance, and Cancer. International journal of molecular sciences (2022) 23(16):204–18. doi: 10.3390/ijms23169313 PMC9409174 36012581

[9] Michelson N Rincon-Torroella J Quiñones-Hinojosa A Greenfield JP. Exploring the role of inflammation in the malignant transformation of low-grade gliomas. J neuroimmunol (2016) 297:132–40. doi: 10.1016/j.jneuroim.2016.05.019 27397086

[10] Szebeni GJ Vizler C Kitajka K Puskas LG. Inflammation and cancer: Extra- and intracellular determinants of tumor-associated macrophages as tumor promoters. Mediators Inflammation (2017) 2017:9294018. doi: 10.1155/2017/9294018 PMC5286482 28197019

[11] Berghoff AS Kiesel B Widhalm G Rajky O Ricken G Wöhrer A. Programmed death ligand 1 expression and tumor-infiltrating lymphocytes in glioblastoma. Neuro-oncology (2015) 17(8):1064–75. doi: 10.1093/neuonc/nou307 PMC4490866 25355681

[12] Lander ES Linton LM Birren B Nusbaum C Zody MC Baldwin J. Initial sequencing and analysis of the human genome. Nature (2001) 409(6822):860–921. doi: 10.1038/35057062 11237011

[13] Choudhari R Sedano MJ Harrison AL Subramani R Lin KY Ramos EI. Long noncoding rnas in cancer: From discovery to therapeutic targets. Adv Clin Chem (2020) 95:105–47. doi: 10.1016/bs.acc.2019.08.003 32122521

[14] Dutta A Roy A Chatterjee S. Long noncoding rnas in cancer immunity: A new avenue in drug discovery. Drug Discovery Today (2021) 26(1):264–72. doi: 10.1016/j.drudis.2020.07.026 32827755

[15] Ponting CP Oliver PL Reik W. Evolution and functions of long noncoding rnas. Cell (2009) 136(4):629–41. doi: 10.1016/j.cell.2009.02.006 19239885

[16] Nukala SB Jousma J Cho Y Lee WH Ong SG. Long non-coding rnas and micrornas as crucial regulators in cardio-oncology. Cell Biosci (2022) 12(1):24. doi: 10.1186/s13578-022-00757-y 35246252 PMC8895873

[17] Cui B Li B Liu Q Cui Y. Lncrna Ccat1 promotes glioma tumorigenesis by sponging mir-181b. J Cell Biochem (2017) 118(12):4548–57. doi: 10.1002/jcb.26116 28475287

[18] Peng Z Liu C Wu M. New insights into long noncoding rnas and their roles in glioma. Mol Cancer (2018) 17(1):61. doi: 10.1186/s12943-018-0812-2 29458374 PMC5817731

[19] Rynkeviciene R Simiene J Strainiene E Stankevicius V Usinskiene J Miseikyte Kaubriene E. Non-coding rnas in glioma. Cancers (Basel) (2018) 11(1):17. doi: 10.3390/cancers11010017 PMC6356972 30583549

[20] Li X Meng Y. Survival analysis of immune-related lncrna in low-grade glioma. BMC Cancer (2019) 19(1):813. doi: 10.1186/s12885-019-6032-3 31419958 PMC6697914

[21] Kang CM Bai HL Li XH Huang RY Zhao JJ Dai XY. The binding of lncrna Rp11-732m18.3 with 14-3-3 B/A accelerates P21 degradation and promotes glioma growth. EBioMedicine (2019) 45:58–69. doi: 10.1016/j.ebiom.2019.06.002 31202814 PMC6642068

[22] Kang CM Zhao JJ Yuan YS Liao JM Yu KW Li WK. Long noncoding rna Rp11-732m18.3 promotes glioma angiogenesis by upregulating vegfa. Front Oncol (2022) 12:873037. doi: 10.3389/fonc.2022.873037 35785190 PMC9247460

[23] Xiang Z Chen X Lv Q Peng X. A novel inflammatory lncrnas prognostic signature for predicting the prognosis of low-grade glioma patients. Front Genet (2021) 12:697819. doi: 10.3389/fgene.2021.697819 34408772 PMC8365518

[24] Guo Q Cheng Y Liang T He Y Ren C Sun L. Comprehensive analysis of lncrna-mrna Co-expression patterns identifies immune-associated lncrna biomarkers in ovarian cancer malignant progression. Sci Rep (2015) 5:17683. doi: 10.1038/srep17683 26631459 PMC4668366

[25] Atianand MK Caffrey DR Fitzgerald KA. Immunobiology of long noncoding rnas. Annu Rev Immunol (2017) 35:177–98. doi: 10.1146/annurev-immunol-041015-055459 PMC6449690 28125358

[26] Sayour EJ McLendon P McLendon R De Leon G Reynolds R Kresak J. Increased proportion of Foxp3+ regulatory T cells in tumor infiltrating lymphocytes is associated with tumor recurrence and reduced survival in patients with glioblastoma. Cancer immunol immunother: CII (2015) 64(4):419–27. doi: 10.1007/s00262-014-1651-7 PMC4774199 25555571

[27] Maghrouni A Givari M Jalili-Nik M Mollazadeh H Bibak B Sadeghi MM. Targeting the pd-1/Pd-L1 pathway in glioblastoma multiforme: Preclinical evidence and clinical interventions. Int Immunopharmacol (2021) 93:107403. doi: 10.1016/j.intimp.2021.107403 33581502

[28] Wang X Guo G Guan H Yu Y Lu J Yu J. Challenges and potential of pd-1/Pd-L1 checkpoint blockade immunotherapy for glioblastoma. J Exp Clin Cancer research: CR (2019) 38(1):87. doi: 10.1186/s13046-019-1085-3 30777100 PMC6380009

[29] Chen B Dragomir MP Yang C Li Q Horst D Calin GA. Targeting non-coding rnas to overcome cancer therapy resistance. Signal Transduct Target Ther (2022) 7(1):121. doi: 10.1038/s41392-022-00975-3 35418578 PMC9008121

[30] Laochareonsuk W Chiengkriwate P Sangkhathat S. Single nucleotide polymorphisms within adducin 3 and adducin 3 antisense Rna1 genes are associated with biliary atresia in Thai infants. Pediatr Surg Int (2018) 34(5):515–20. doi: 10.1007/s00383-018-4243-3 29508064

[31] Zeng S Zhou C Yang DH Xu LS Yang HJ Xu MH. Lef1-As1 is implicated in the malignant development of glioblastoma *Via* sponging mir-543 to upregulate En2. Brain Res (2020) 1736:146781. doi: 10.1016/j.brainres.2020.146781 32184164

[32] Peng WX He RZ Zhang Z Yang L Mo YY. Linc00346 promotes pancreatic cancer progression through the ctcf-mediated myc transcription. Oncogene (2019) 38(41):6770–80. doi: 10.1038/s41388-019-0918-z 31391552

[33] Ye T Ding W Wang N Huang H Pan Y Wei A. Long noncoding rna Linc00346 promotes the malignant phenotypes of bladder cancer. Biochem Biophys Res Commun (2017) 491(1):79–84. doi: 10.1016/j.bbrc.2017.07.045 28705739

[34] Hohman TJ Koran ME Thornton-Wells TA. Genetic variation modifies risk for neurodegeneration based on biomarker status. Front Aging Neurosci (2014) 6:183. doi: 10.3389/fnagi.2014.00183 25140149 PMC4121544

[35] Son HY Hwangbo Y Yoo SK Im SW Yang SD Kwak SJ. Genome-wide association and expression quantitative trait loci studies identify multiple susceptibility loci for thyroid cancer. Nat Commun (2017) 8:15966. doi: 10.1038/ncomms15966 28703219 PMC5511346

[36] Ye N Rao S Du T Hu H Liu Z Shen Y. Intergenic variants may predispose to major depression disorder through regulation of long non-coding rna expression. Gene (2017) 601:21–6. doi: 10.1016/j.gene.2016.11.041 27940106

[37] Maciukiewicz M Marshe VS Hauschild AC Foster JA Rotzinger S Kennedy JL. Gwas-based machine learning approach to predict duloxetine response in major depressive disorder. J Psychiatr Res (2018) 99:62–8. doi: 10.1016/j.jpsychires.2017.12.009 29407288

[38] Li S Li Y Deng B Yan J Wang Y. Identification of the differentially expressed genes involved in the synergistic neurotoxicity of an hiv protease inhibitor and methamphetamine. Curr HIV Res (2019) 17(4):290–303. doi: 10.2174/1570162x17666190924200354 31550215

[39] He Y Li X Meng Y Fu S Cui Y Shi Y. A prognostic 11 long noncoding rna expression signature for breast invasive carcinoma. J Cell Biochem (2019) 120(10):16692–702. doi: 10.1002/jcb.28927 31095790

[40] Wang X Zhou J Xu M Yan Y Huang L Kuang Y. A 15-lncrna signature predicts survival and functions as a cerna in patients with colorectal cancer. Cancer Manag Res (2018) 10:5799–806. doi: 10.2147/cmar.S178732 PMC6248371 30510449

[41] Fu X Wang D Shu T Cui D Fu Q. Lncrna Nr2f2-As1 positively regulates Cdk4 to promote cancer cell proliferation in prostate carcinoma. Aging Male (2020) 23(5):1073–9. doi: 10.1080/13685538.2019.1670157 31566058

[42] Zhang S Zhang X Sun Q Zhuang C Li G Sun L. Lncrna Nr2f2-As1 promotes tumourigenesis through modulating Bmi1 expression by targeting mir-320b in non-small cell lung cancer. J Cell Mol Med (2019) 23(3):2001–11. doi: 10.1111/jcmm.14102 PMC6378175 30592135

[43] Shiraishi D Fujiwara Y Komohara Y Mizuta H Takeya M. Glucagon-like peptide-1 (Glp-1) induces M2 polarization of human macrophages *Via* Stat3 activation. Biochem Biophys Res Commun (2012) 425(2):304–8. doi: 10.1016/j.bbrc.2012.07.086 22842565

[44] Komohara Y Horlad H Ohnishi K Ohta K Makino K Hondo H. M2 Macrophage/Microglial cells induce activation of Stat3 in primary central nervous system lymphoma. J Clin Exp Hematop (2011) 51(2):93–9. doi: 10.3960/jslrt.51.93 22104307

[45] Popovic A Jaffee EM Zaidi N. Emerging strategies for combination checkpoint modulators in cancer immunotherapy. J Clin Invest (2018) 128(8):3209–18. doi: 10.1172/jci120775 PMC6063475 30067248

