# Supplementary material for: A novel inflammation-related lncRNAs prognostic signature identifies LINC00346 in promoting proliferation, migration, and immune infiltration of glioma
Source: Front Immunol. 2022 Oct 13;13:810572. doi: 10.3389/fimmu.2022.810572 (PMC9609424; doi:10.3389/fimmu.2022.810572)
Supplement: Supplementary file 1 [file DataSheet_1.docx]

**Supplementary information**


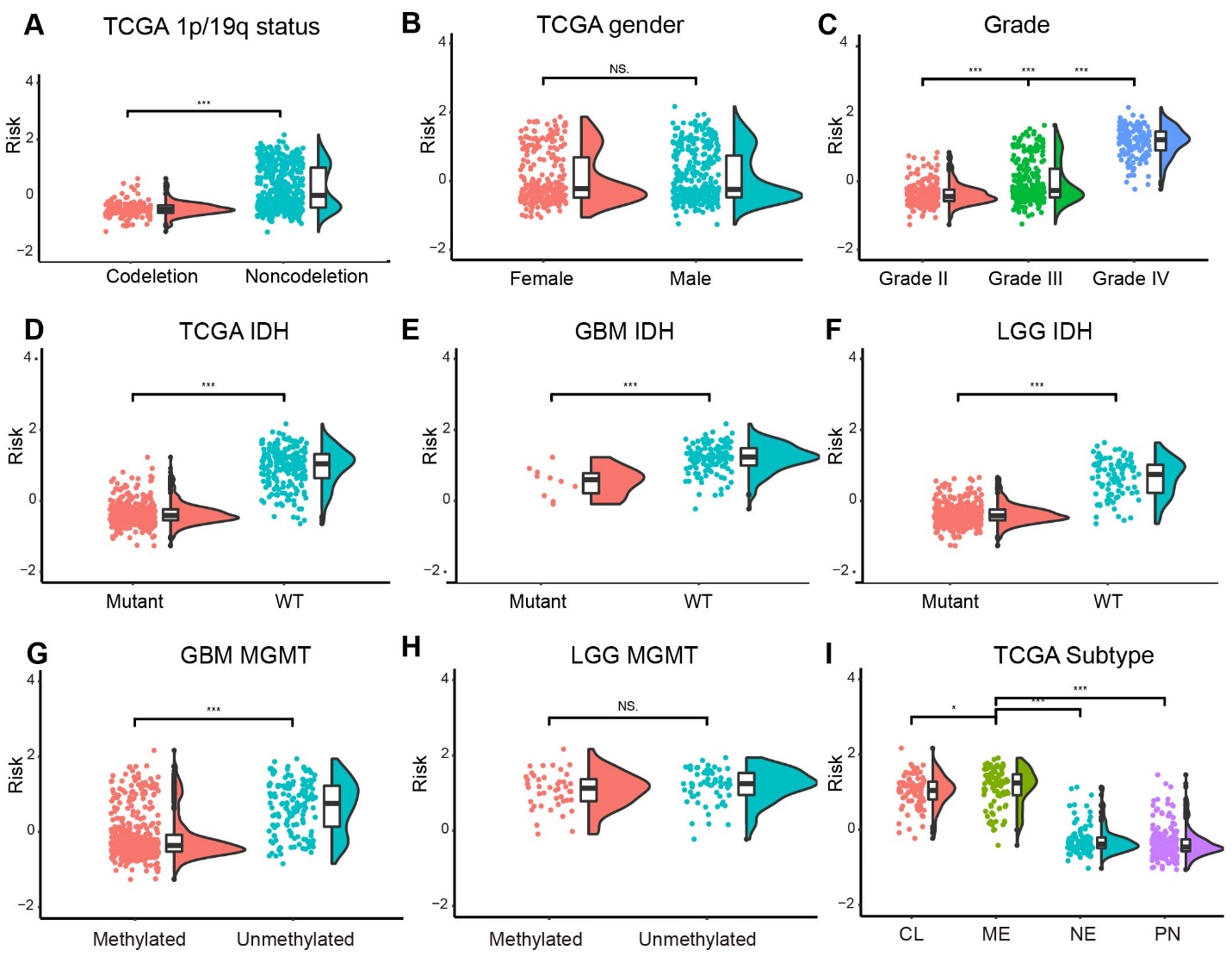


Fig. S1. The risk scores for different subgroups in TCGA glioma cohorts. (A) 1p/19q status in TCGA dataset. (B) Female and male patients in TCGA dataset. (C) WHO grade in TCGA dataset. (D) *IDH* status in TCGA dataset. (E) *IDH* status in TCGA GBM patients. (F) *IDH* status in TCGA LGG patients. (G) *MGMT* promoter methylation in the TCGA GBM patients. (H) *MGMT* promoter methylation in the TCGA LGG patients. (I) Molecular subtypes of gliomas in TCGA dataset. CL: Classical subtype, ME: Mesenchymal subtype, NE: Neuronal subtype, PN: Proneuronal subtype.


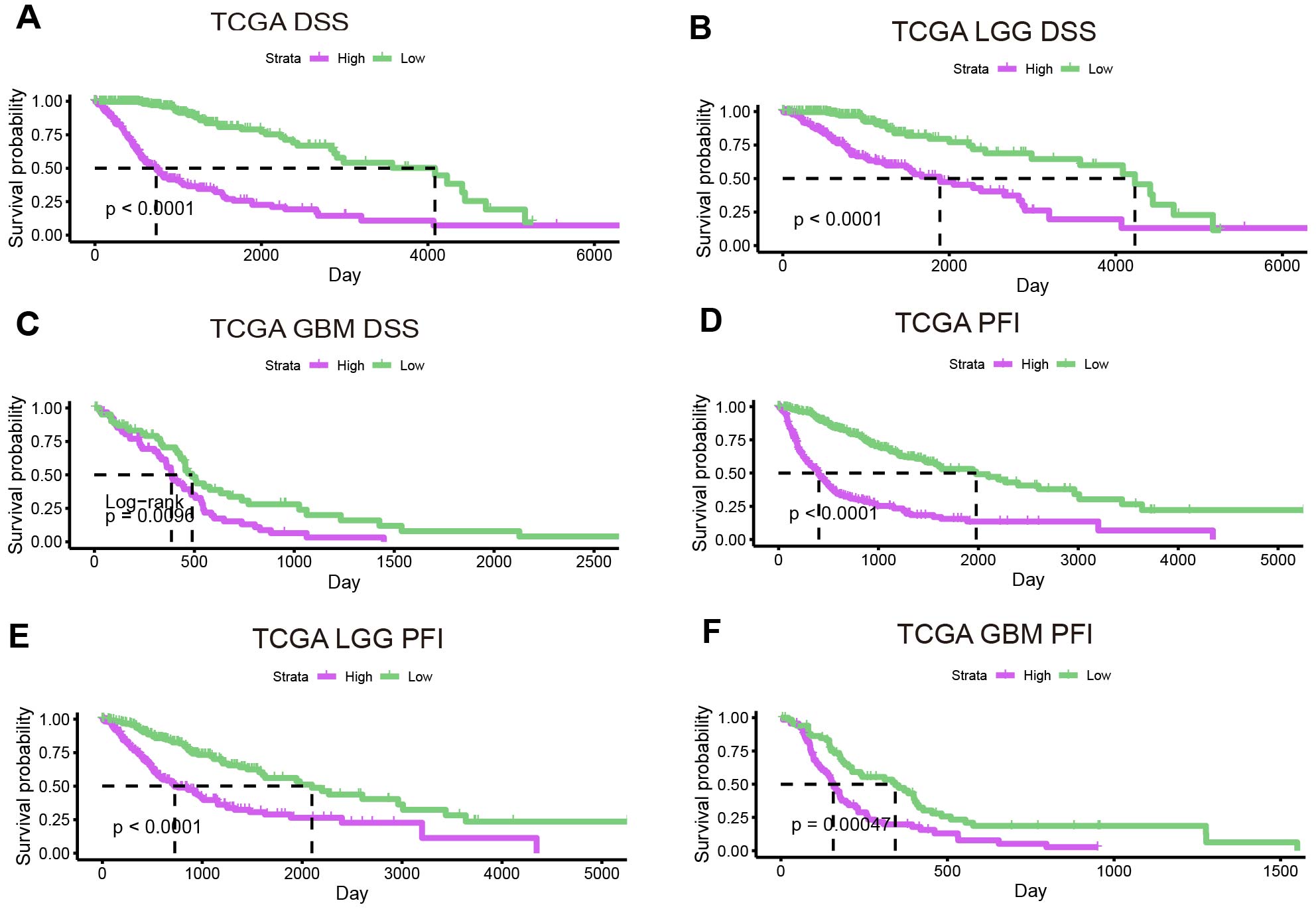


Fig. S2. Establishment and verification the prognostic value of the risk score model. Differences in DSS and PFI between high and low risk groups among total, LGG, and GBM patients in the TCGA dataset (*P* < 0.0001).


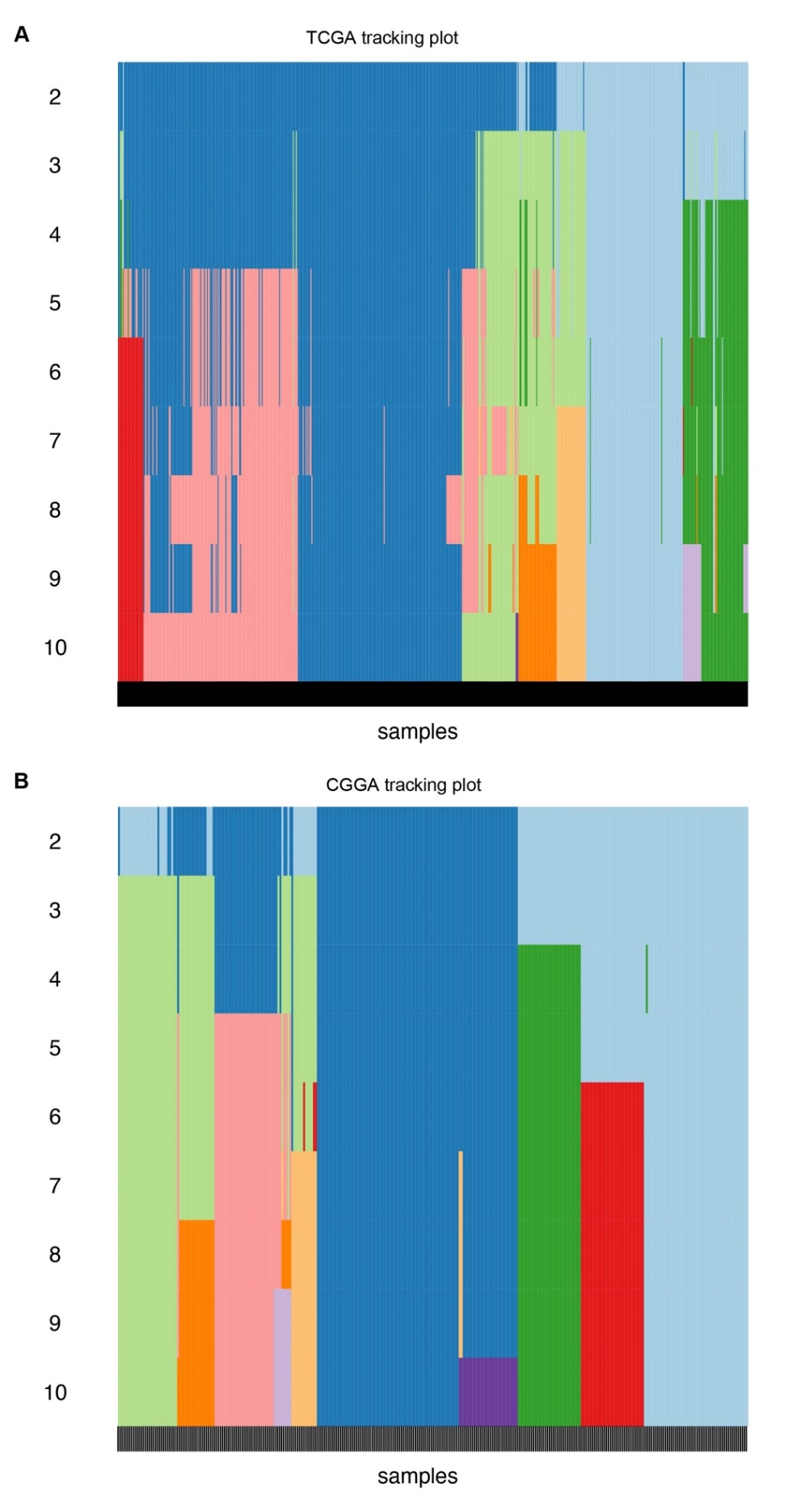


Fig. S3. Tracking plot in the TCGA and CGGA datasets.


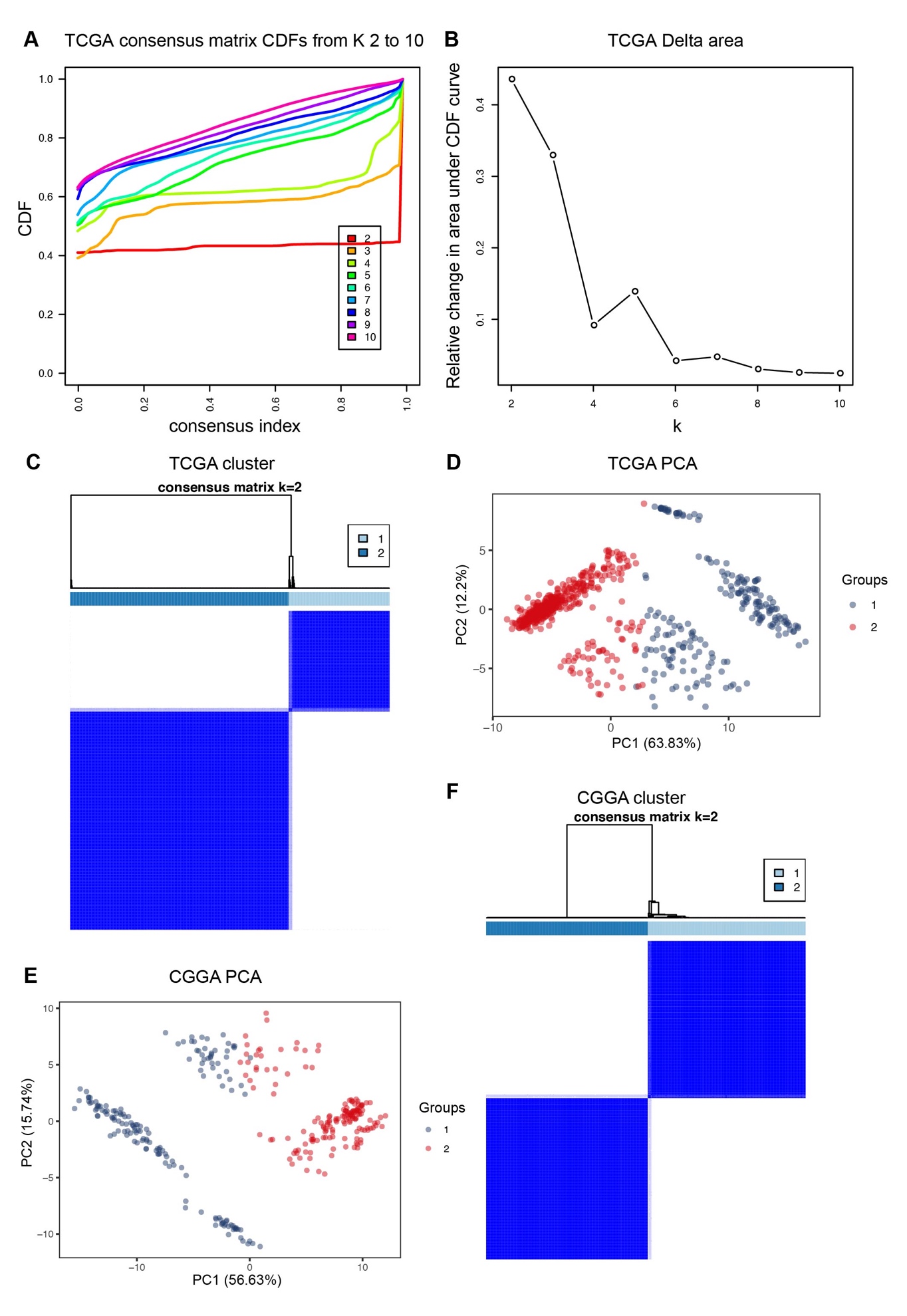


Fig. S4. Consensus clustering. (A) Consensus matrix CDFs in the TCGA dataset. (B) Relative change in the area under curve of CDF. (C) Heatmap of consensus matrix from K = 2 in the TCGA dataset. (D) Principal component analysis (PCA) in the TCGA dataset. (E) PCA in the CGGA dataset. (F) Heatmap of consensus matrix from K = 2 in the CGGA dataset.


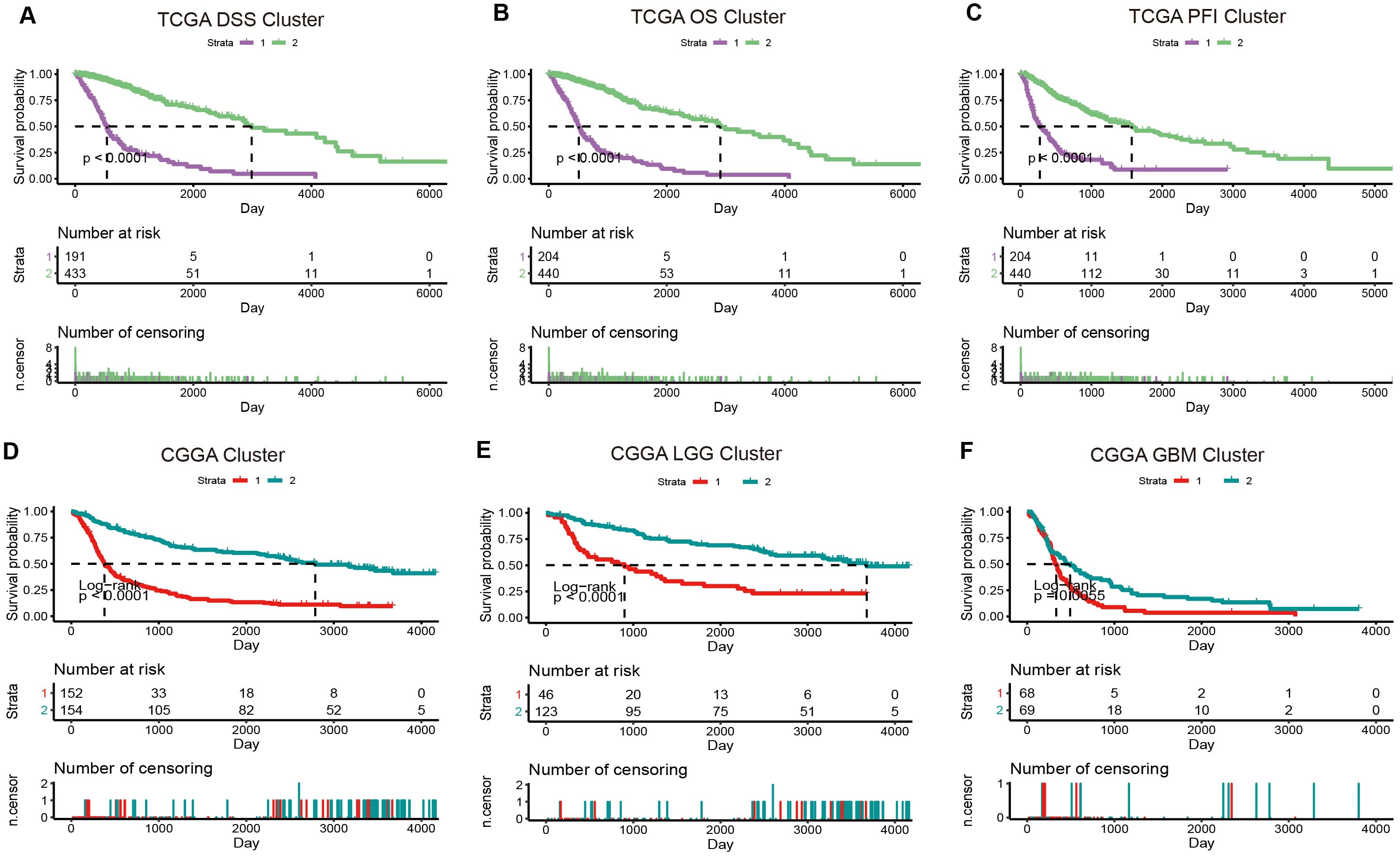


Fig. S5. Differences in DSS and PFI between cluster 1 and cluster 2 among total, LGG, and GBM patients in the TCGA and CCGA datasets.


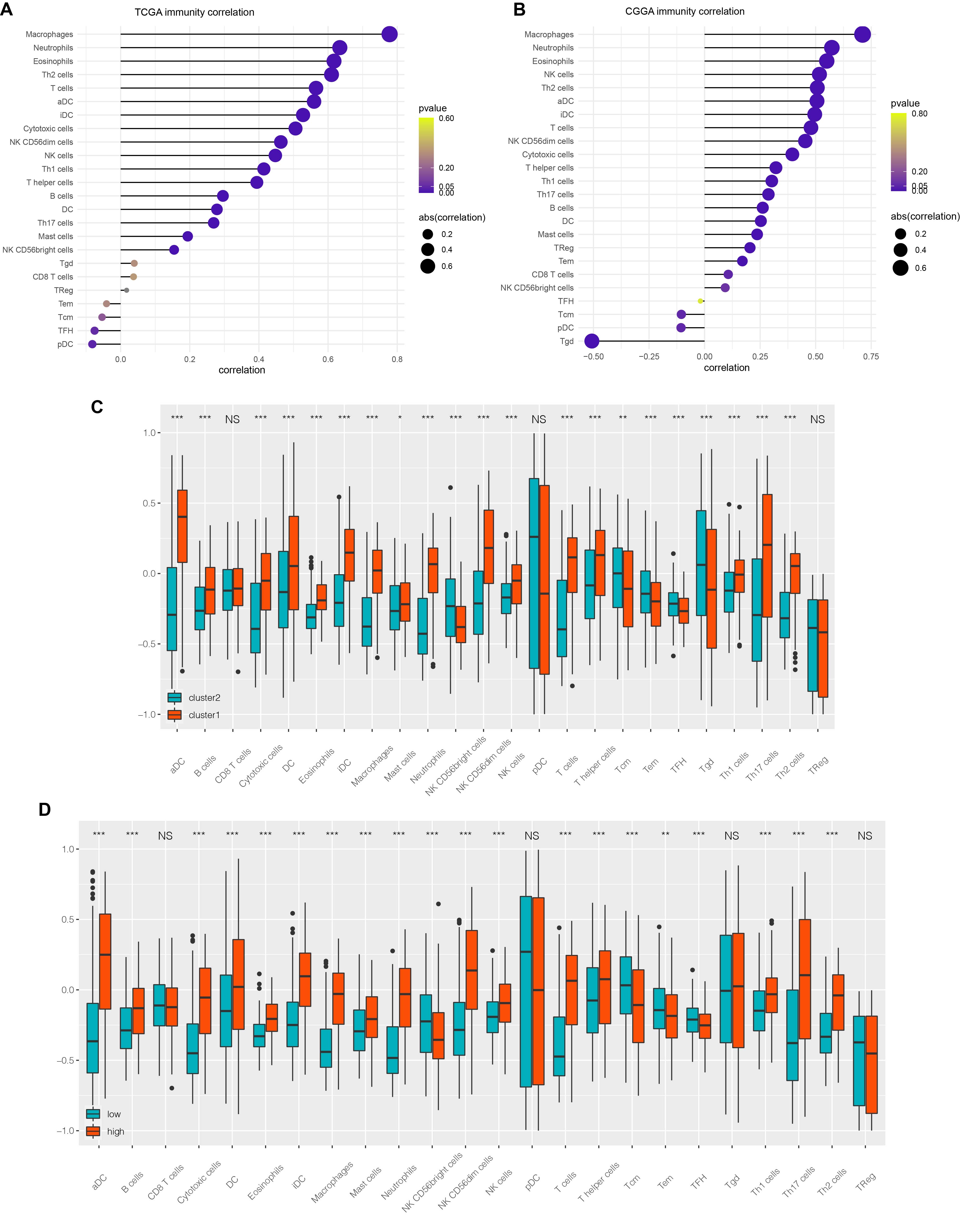


Fig. S6. (A, B) Correlation between immune cell types and LINC00346 in the TCGA and CGGA datasets. (C) Immune cell composition in distinct clusters in TCGA dataset . (D) Immune cell composition in distinct risk groups in TCGA dataset .


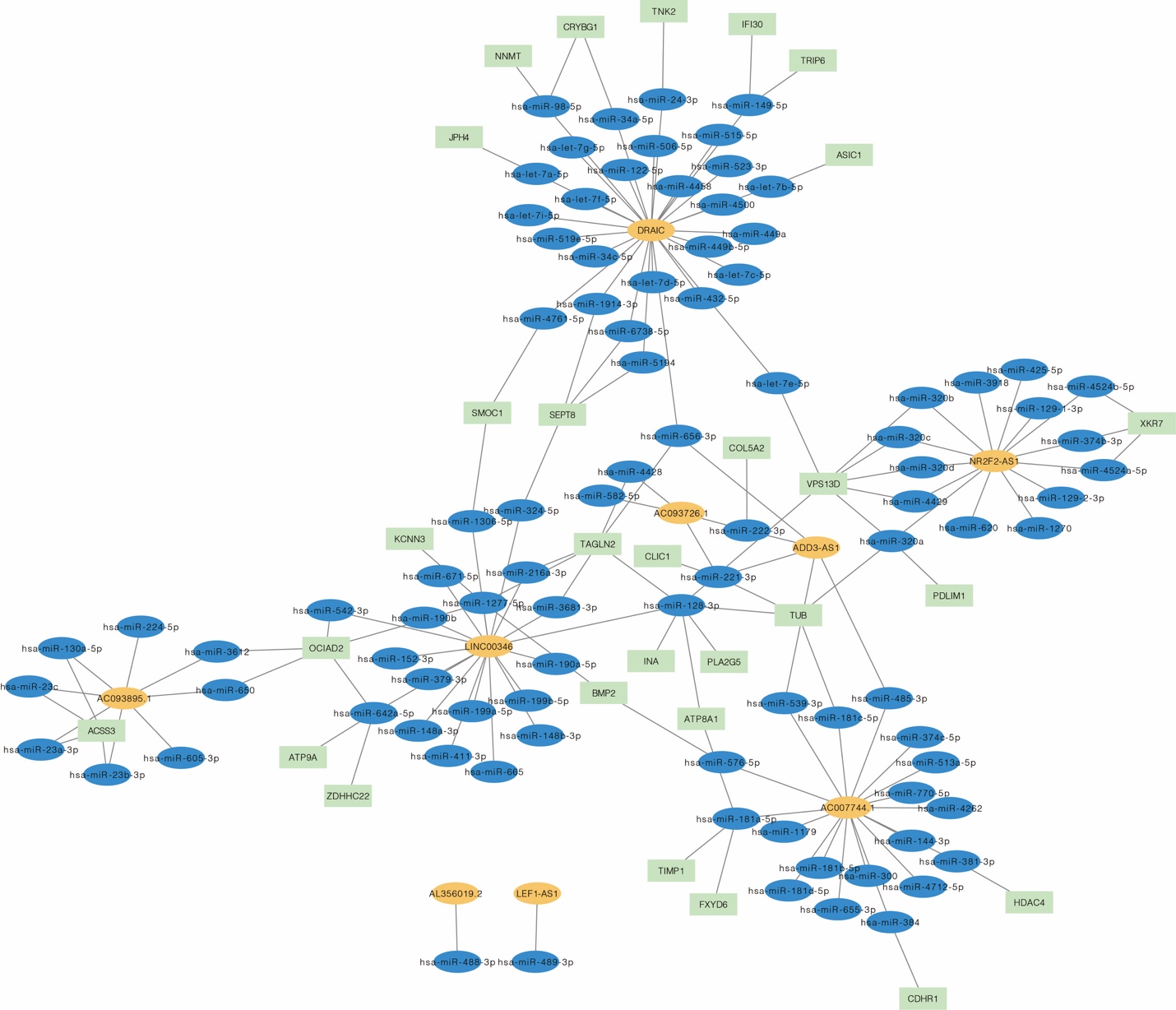


Fig S7. In total, 9 lncRNAs (yellow), 93 miRNAs (blue) and 29-relatived mRNAs (green) were used to construct the lncRNA-miRNA-mRNA ceRNA network.


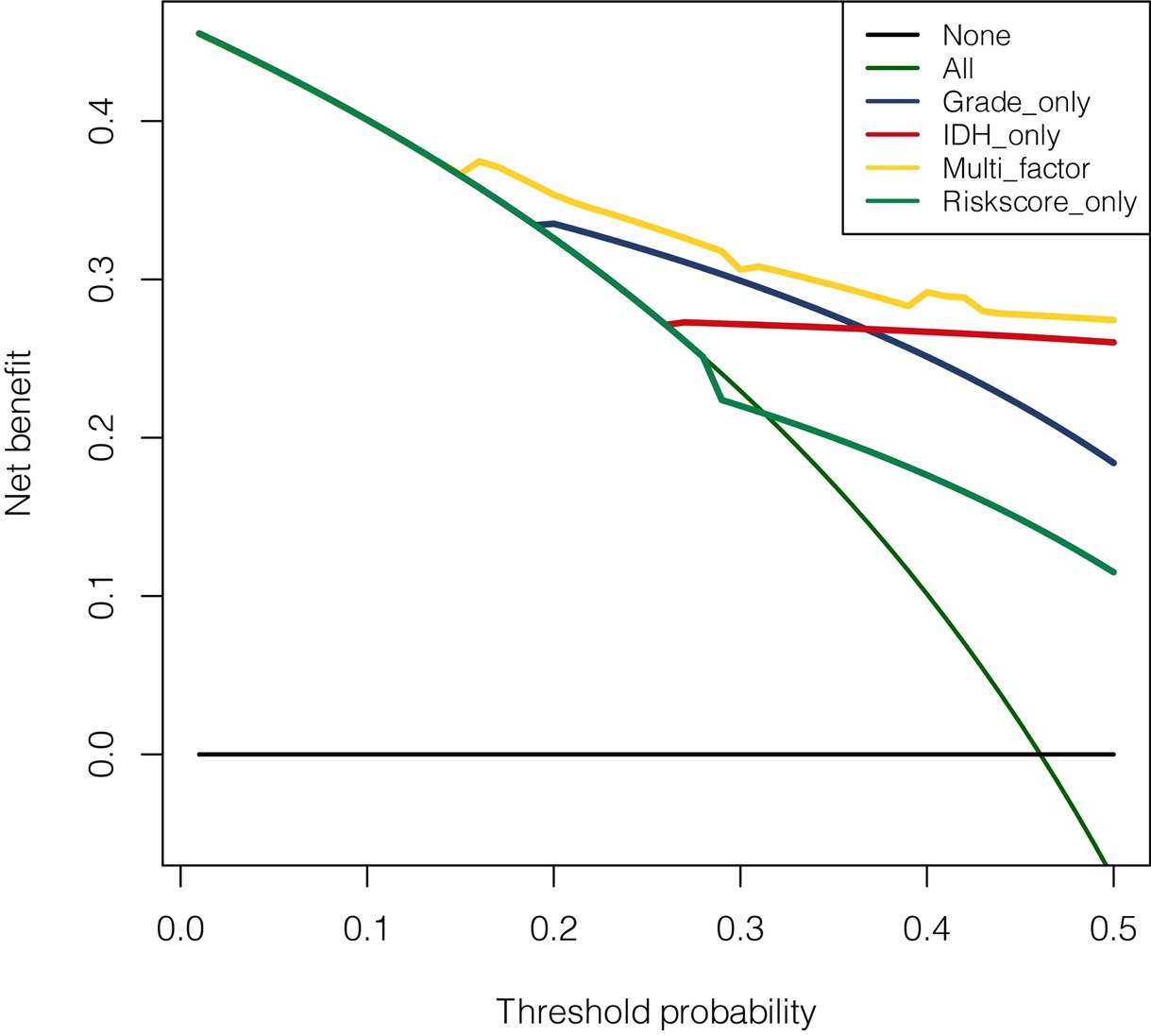


Fig S8. Decision Curve Analysis (DCA) results in 5-year survival predictions, showing that the multi factor model prognostic prediction based on the lncRNAs added more net benefit than the “IDH only”, “Grade only” strategies in the TCGA datasets.

**
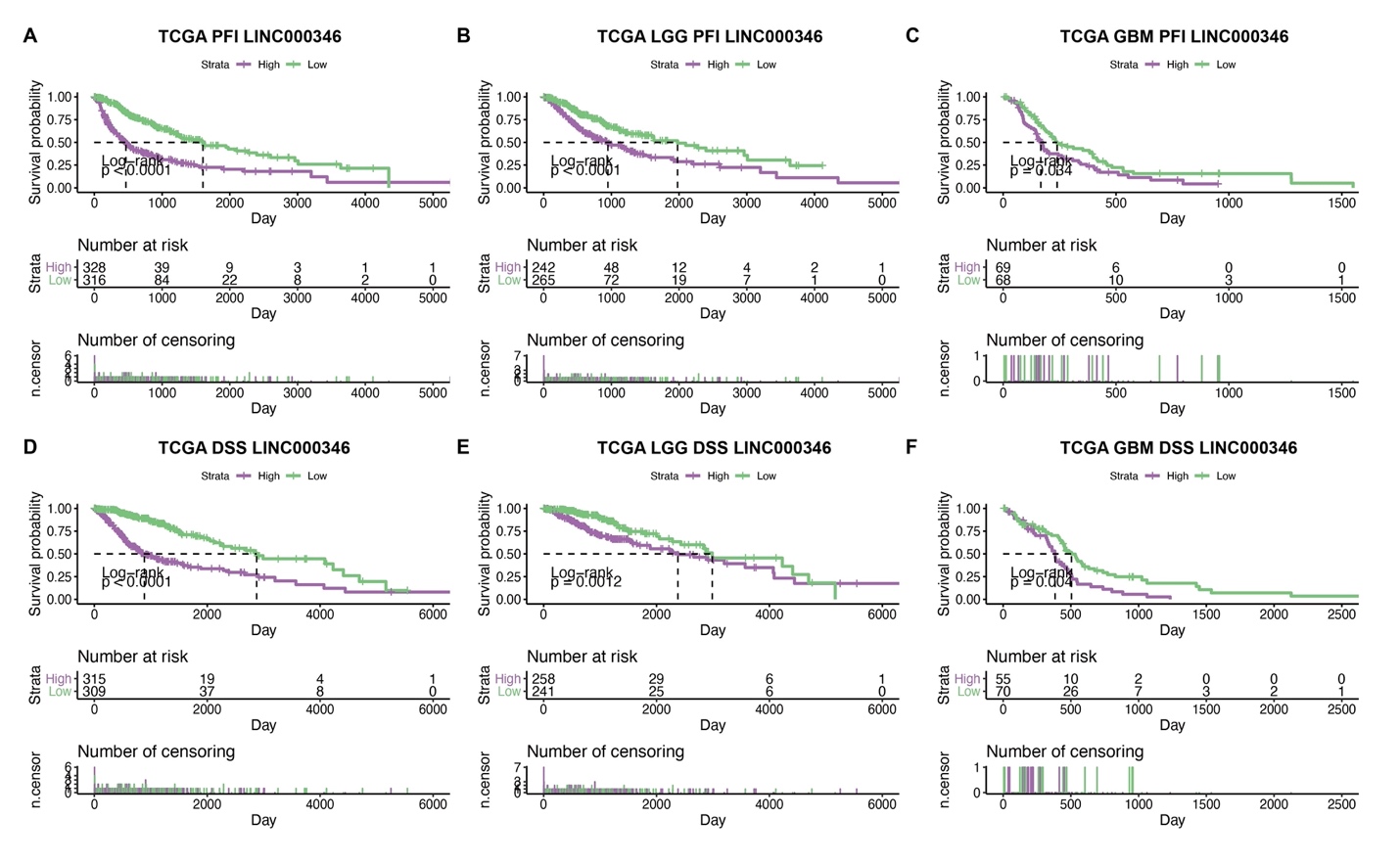
**

Fig. S9. Establishment and verification of a risk score model based on LINC00346 expression. (A-C) Clinical survival outcomes of (A) sum, (B) LGG and (C) GBM patients in high or low-risk groups using PFI as the endpoint in the TCGA dataset (p < 0.05). (D-F) Clinical survival outcomes of (D) total, (E) LGG and (F) GBM patients in high or low-risk groups using DSS as the endpoint in the TCGA dataset (p < 0.05).


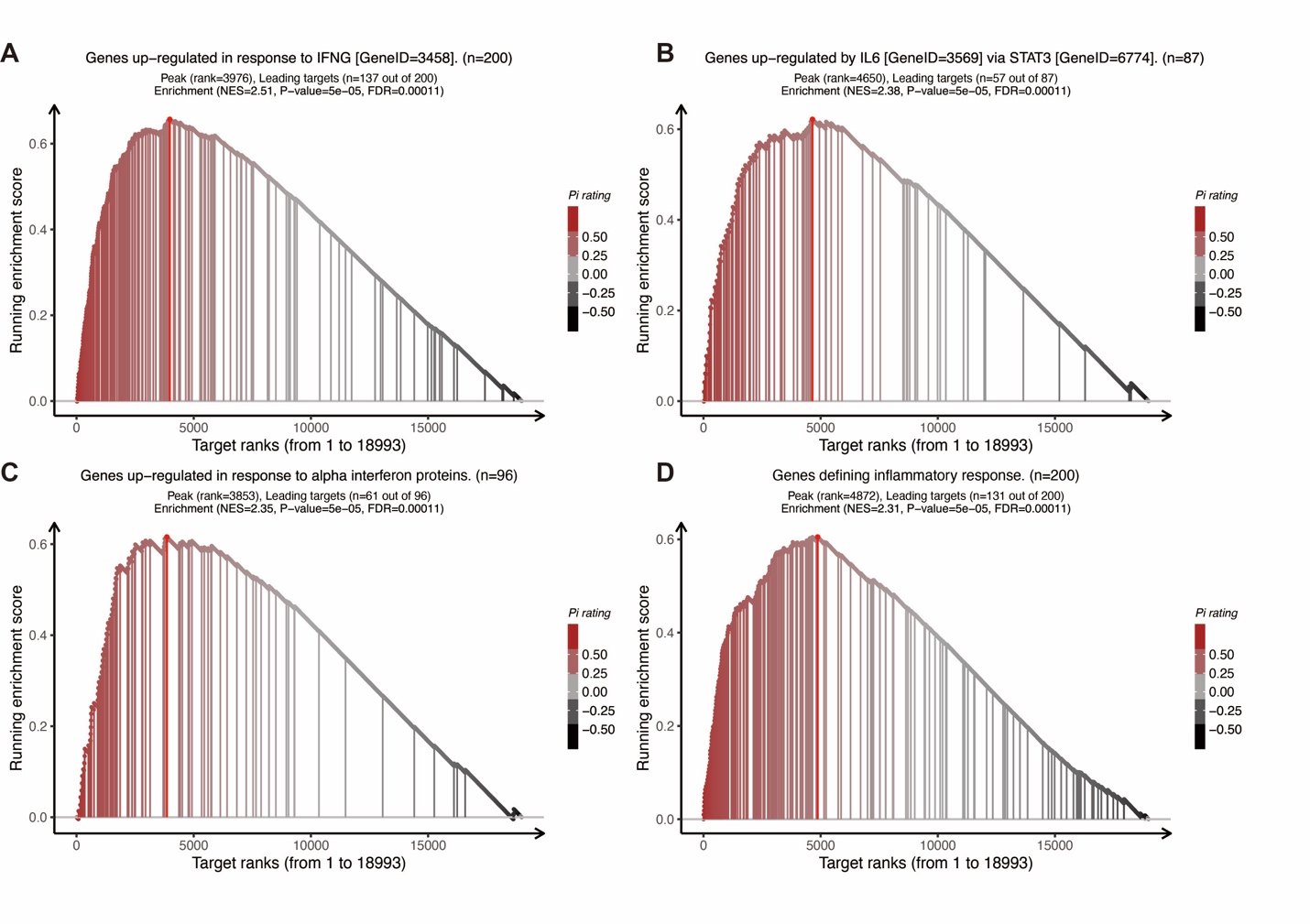


Fig. S10. Gene Set Enrichment Analysis (GSEA) showed that the high-expression LINC00346 subset was mainly associated with important inflammation-related hallmarks.


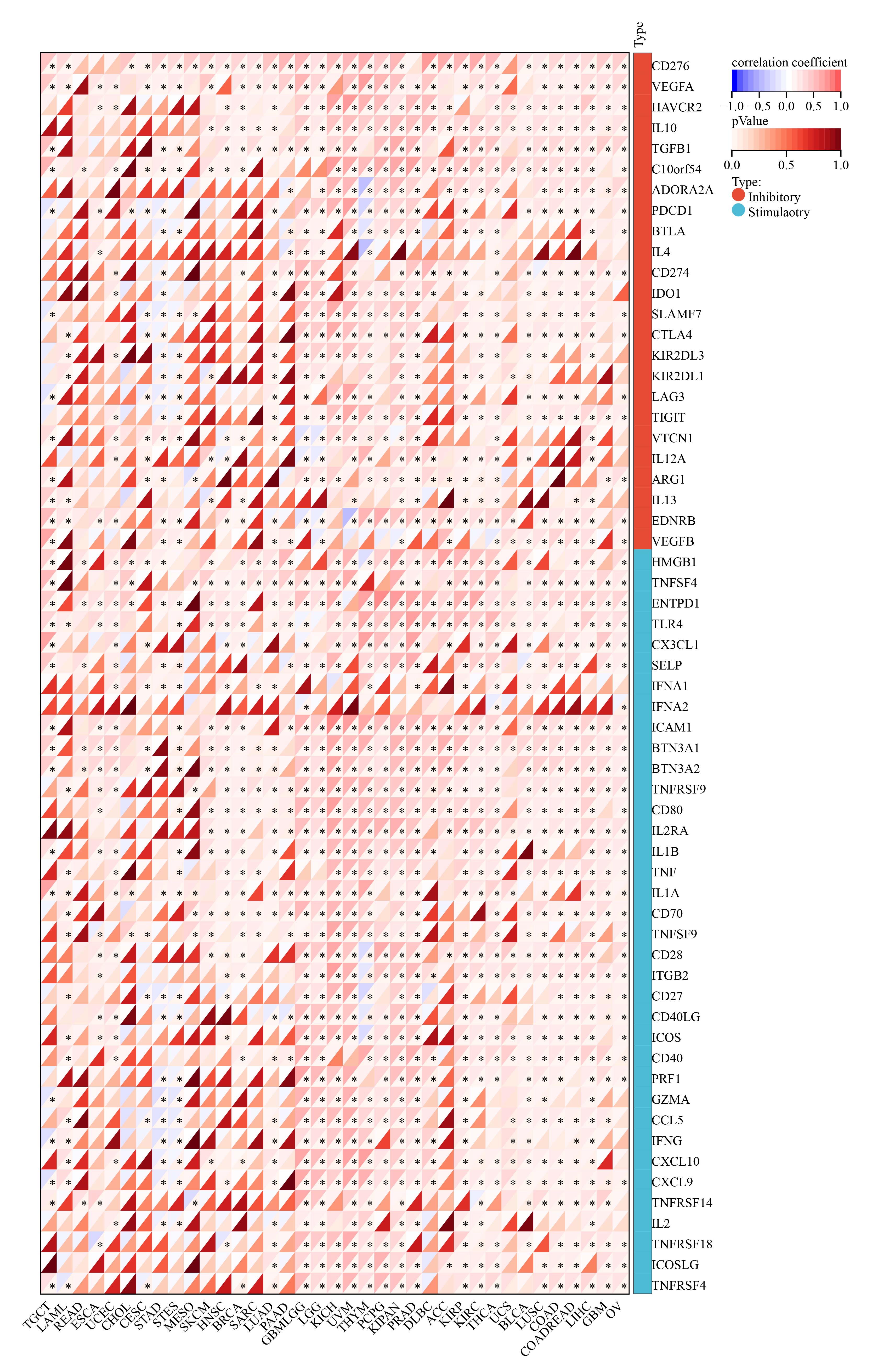


Fig. S11. Correlation between LINC00346 expression and immune checkpoint gene levels in pan-cancer.


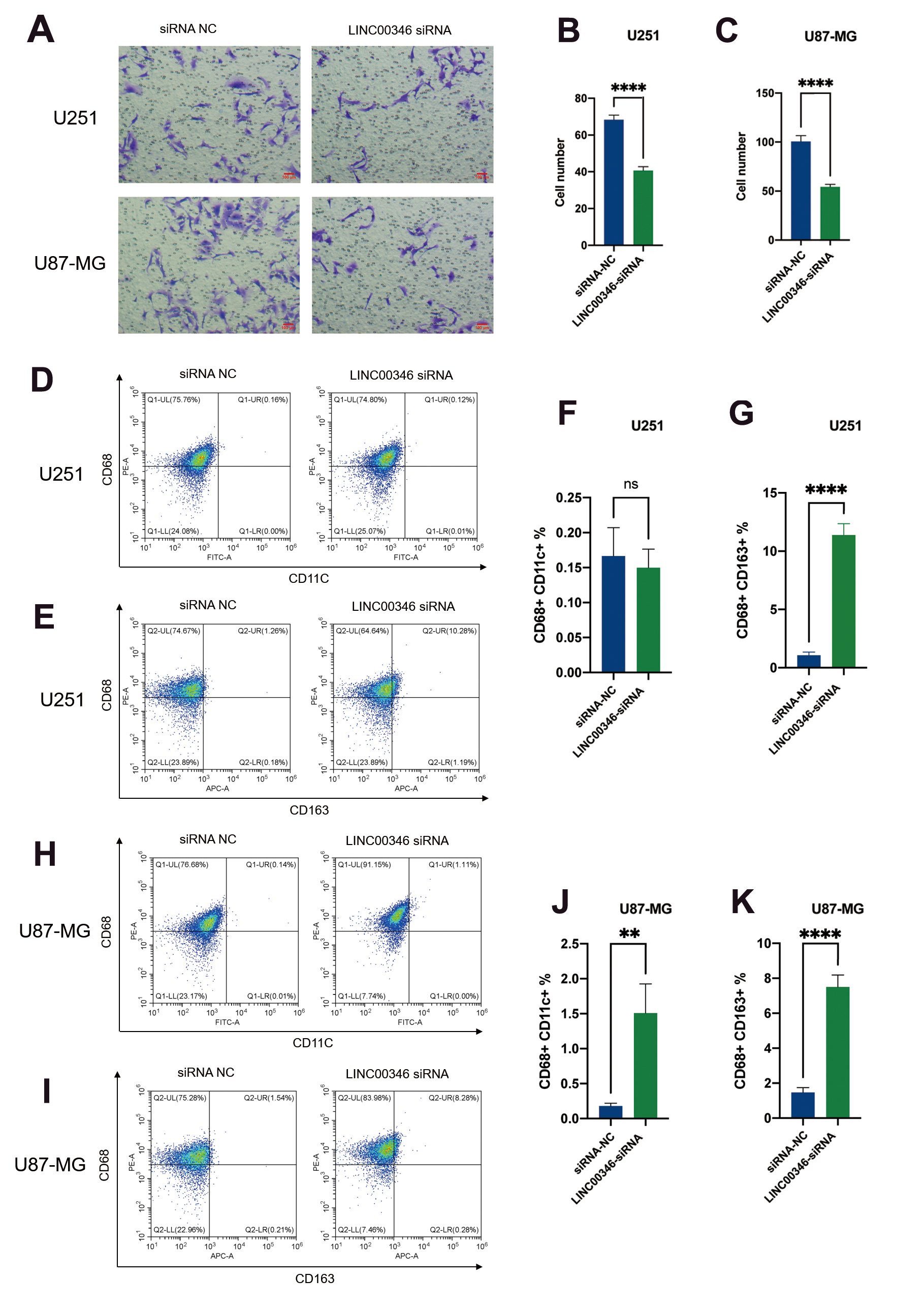


Fig. S12. LINC00346 affected the migration of macrophages in glioma. U87 or U251 cells were transfected with siRNA for 24 hours, then they were co-culture with macrophages HMC3. (A-C) Transwell assay was used to detect the migration of macrophage.
